# Ancestral aneuploidy and stable chromosomal duplication resulting in differential genome structure and gene expression control in trypanosomatid parasites

**DOI:** 10.1101/gr.278550.123

**Published:** 2024-03

**Authors:** João L. Reis-Cunha, Samuel A. Pimenta-Carvalho, Laila V. Almeida, Anderson Coqueiro-dos-Santos, Catarina A. Marques, Jennifer A. Black, Jeziel Damasceno, Richard McCulloch, Daniella C. Bartholomeu, Daniel C. Jeffares

**Affiliations:** 1 York Biomedical Research Institute, Department of Biology, University of York, York YO10 5DD, United Kingdom;; 2 Instituto de Ciências Biológicas, Departamento de Parasitologia, Universidade Federal de Minas Gerais, Belo Horizonte, 31270-901, Brazil;; 3 The Wellcome Centre for Integrative Parasitology, School of Infection and Immunity, University of Glasgow, Glasgow G12 8TA, United Kingdom;; 4 Faculdade de Medicina de Ribeirão Preto, Universidade de São Paulo, Ribeirão Preto, 14049-900, Brazil

## Abstract

Aneuploidy is widely observed in both unicellular and multicellular eukaryotes, usually associated with adaptation to stress conditions. Chromosomal duplication stability is a tradeoff between the fitness cost of having unbalanced gene copies and the potential fitness gained from increased dosage of specific advantageous genes. Trypanosomatids, a family of protozoans that include species that cause neglected tropical diseases, are a relevant group to study aneuploidies. Their life cycle has several stressors that could select for different patterns of chromosomal duplications and/or losses, and their nearly universal use of polycistronic transcription increases their reliance on gene expansion/contraction, as well as post-transcriptional control as mechanisms for gene expression regulation. By evaluating the data from 866 isolates covering seven trypanosomatid genera, we have revealed that aneuploidy tolerance is an ancestral characteristic of trypanosomatids but has a reduced occurrence in a specific monophyletic clade that has undergone large genomic reorganization and chromosomal fusions. We have also identified an ancient chromosomal duplication that was maintained across these parasite's speciation, named collectively as the trypanosomatid ancestral supernumerary chromosome (TASC). TASC has most genes in the same coding strand, is expressed as a disomic chromosome (even having four copies), and has increased potential for functional variation, but it purges highly deleterious mutations more efficiently than other chromosomes. The evidence of stringent control over gene expression in this chromosome suggests that these parasites have adapted to mitigate the fitness cost associated with this ancient chromosomal duplication.

Aneuploidy, the presence of an unequal number of chromosomal copies, is widely found in nature, being observed in protozoans, yeast, plants, and animals. The stability of chromosomal duplications was shown to be a tradeoff between the fitness cost of having unbalanced gene copies and the potential fitness gained from an increased dosage of specific advantageous genes (Pompei and Cosentino Lagomarsino 2023). Most aneuploidy studies have focused on cancer or yeast, in which aneuploidy has been linked with loss of cell cycle control, survival in stressful conditions, and adaptability (Gilchrist and Stelkens 2019; Ben-David and Amon 2020). Aneuploidies are the most common genomic alteration in cancer, present in ∼90% of solid and ∼50% of hematopoietic tumors (for review, see Ben-David and Amon 2020), and removal of trisomic chromosomes was recently shown to compromise cancer growth (Girish et al. 2023). Although aneuploid cancer cells may also have reduced growth rates, particular combinations of chromosomal duplication can be advantageous under specific conditions (Williams et al. 2008; Graham et al. 2017; Hwang et al. 2021; Pompei and Cosentino Lagomarsino 2023) and can promote chemotherapy resistance (Lukow et al. 2021). Specific chromosomal duplications were also linked to resistance against antifungal agents in yeast (Selmecki et al. 2008, 2009; Chen et al. 2012, 2015). Hence, aneuploidies have been shown to provide a rapid and often transient solution to stress conditions, emerging more frequently than other genetic alterations, such as single-nucleotide polymorphisms (SNPs), short insertion/deletions and gene copy number variants (CNVs) (Yona et al. 2012; Pompei and Cosentino Lagomarsino 2023).

Trypanosomatidae, a family of obligatory single-celled parasites from the kinetoplastid order (phylum Euglenozoa), are an interesting group to study aneuploidies. Throughout their life cycles, they are exposed to several stressors, to which chromosome instability (CIN), having a persistent rate of chromosome duplication and loss that generates intra-population variation of chromosomal copies, would be beneficial. Some examples are evasion of the host immune response and, for heteroxenous parasites, environmental changes encountered when moving between insect vectors and vertebrate hosts. These core features of the trypanosomatid life cycle likely require widespread changes in gene expression, which may be facilitated by selected chromosomal expansion patterns (Mannaert et al. 2012; Dumetz et al. 2017; Reis-Cunha et al. 2017). Moreover, trypanosomatid biology is unusual, and one reason for aneuploidy-driven gene expression control could be linked to their near universal use of polycistronic transcription, limiting their capacity to alter the transcription of individual genes via promoters and instead increasing their reliance on mechanisms of gene expansion and contraction and on post-transcriptional control mechanisms (Teixeira et al. 2012; Dumetz et al. 2017; Iantorno et al. 2017; Prieto Barja et al. 2017; Damasceno et al. 2021).

The focus of aneuploidy studies in trypanosomatids has largely been directed toward *Leishmania*, *Trypanosoma cruzi*, and *Trypanosoma brucei*. In humans and animals, these pathogenic trypanosomatids cause devastating neglected tropical diseases (NTDs), which disproportionately impose severe health and economic burdens upon developing countries (Burza et al. 2018; Kennedy 2019; https://www.paho.org/en/topics/chagas-disease). These three pathogens represent a relevant group to study aneuploidy owing to contrasting evidence for its presence across the different clades. Notably, aneuploidy is common among *Leishmania* species (Rogers et al. 2011; Dumetz et al. 2017; Prieto Barja et al. 2017) and *T. cruzi* discrete typing units (DTUs) (Reis-Cunha et al. 2015, 2018) but appears to be rare in *T. brucei* (Almeida et al. 2018; Cosentino et al. 2021). Aneuploidy has also been evaluated in other trypanosomatids (Flegontov et al. 2016; Albanaz et al. 2021; Davey et al. 2021), although our understanding of this phenomenon across the full trypanosomatid clade is limited.

Aneuploidies have been widely studied in the *Leishmania* genus, in which they have been documented in all species, likely arising stochastically in individual cells within a population by CIN, in a phenomenon known as “mosaic aneuploidy” (Rogers et al. 2011; Sterkers et al. 2011; Lachaud et al. 2014; Black et al. 2023). Recent single-cell sequencing experiments have confirmed the coexistence of a mosaic diversity of karyotypes within *Leishmania* clonal lines, showing that bulk DNA sequencing represents the most common patterns of somy in a population but underestimates the total variation in somy at the individual cell level (Negreira et al. 2022). Notably, the pattern of chromosomal expansions in *Leishmania* varies greatly in long-term culture (Dumetz et al. 2017) and to some extent in the mammalian host*,* as confirmed by DNA FISH (Prieto Barja et al. 2017). The presence of a polysomy in Chromosome 8 in long-term mammalian infection, undetected in the original culture isolate, suggests that different environments may favor specific combinations of chromosomal duplications (Dumetz et al. 2017). In fact, preadapted *Leishmania donovani* karyotypes were further modulated de novo under strong antimony pressure, potentially providing resistance in vitro (Negreira et al. 2023). Aneuploidy, more specifically chromosome duplication followed by loss, was also associated with loss of heterozygosity (LoH) in *Leishmania* (Prieto Barja et al. 2017). This process, named haplotype selection, enables the protozoan to eliminate potentially disadvantageous chromosomal copies and alleles without sexual recombination, and indicates that aneuploidy is a relevant process in *Leishmania*’s survival and evolution. Chromosomal duplications were also shown to directly impact gene expression, in which chromosomes with extra copies were highly expressed compared with disomic counterparts, with the sole exception of *Leishmania* Chromosome 31 (Leish Chr 31). Leish Chr 31 was found to be expressed as disomic, despite having about four copies (Dumetz et al. 2017), suggesting that specific expression control mechanisms operate in this chromosome. Both the basis for this expression control in Leish Chr 31 and its persistent supernumerary status across all *Leishmania* species remain unexplained.

To date, analysis of aneuploidy beyond *Leishmania* has been focused on genome surveys in *T. brucei* strains and *T. cruzi* DTUs (Reis-Cunha et al. 2015, 2018; Almeida et al. 2018), suggesting that the process is rare in the former and somewhat more common in the latter. Although some examples of LoH and trisomy have been documented in *T. brucei*, such as during the adaptation of isolates to culture (Cosentino et al. 2021; Mulindwa et al. 2021), its prevalence and impact have not been explored to the same depth as in *Leishmania*. More importantly, it is unclear when the capacity for aneuploidy arose during trypanosomatid evolution. In the present work, we evaluated aneuploidy prevalence across the trypanosomatid clade, examining isolates and clades with near-chromosome-level-assembled reference genomes and availability of whole-genome sequencing (WGS) read libraries. We aimed to identify if aneuploidy is an ancestral or derived feature in these organisms, as well as what is the long-term impact of chromosomal duplication during the evolution of these parasites.

## Results

### Aneuploidy is an ancestral characteristic in trypanosomatids, and consistent chromosomal expansions are observed in the majority of clades

To evaluate the occurrence of aneuploidy across trypanosomatids, we determined the chromosome copy number variation (CCNV) in a representative data set containing 866 isolates from seven genera: *Crithidia*, *Endotrypanum*, *Leishmania*, *Leptomonas*, *Paratrypanosoma*, *Porcisia*, and *Trypanosoma* (Supplemental Tables S1–S3), with at least five isolates from each genera, besides *Paratrypanosoma*. This gave us an unprecedented resolution not only to comprehend the presence/absence of chromosomal expansions but also to identify their patterns and commonalities within and across clades.

**Figure 1.**
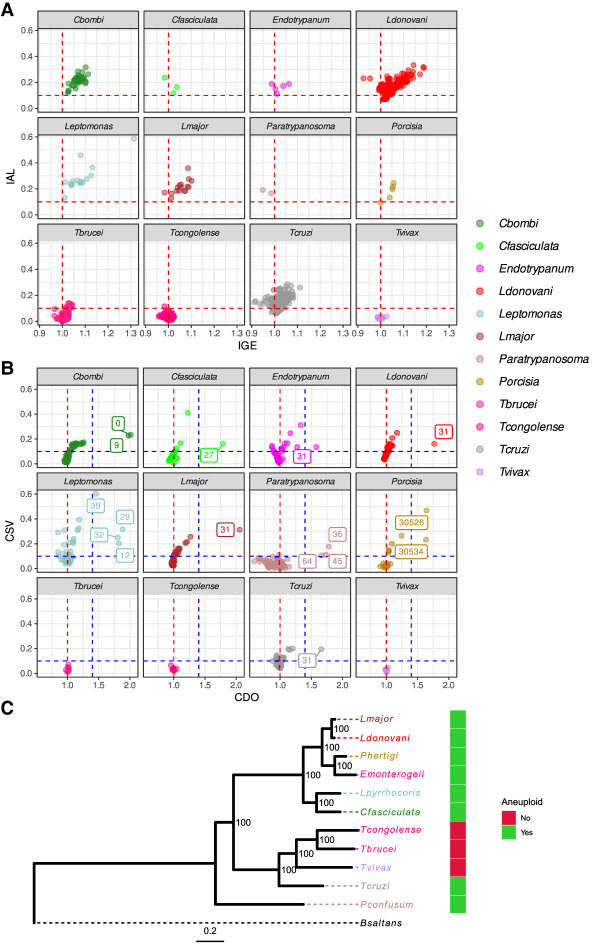
Aneuploidy is ancestral and variable in trypanosomatids. (*A*) Evaluation of CCNV among trypanosomatid isolates. Each dot corresponds to an isolate. The *x*- and *y*-axes represent, respectively, IGE and IAL for each isolate. The vertical red dashed line in the *x*-value of one represents the expected copy number for euploid samples, with one copy for each haploid genome copy. The horizontal red dashed line corresponds to the IAL of 0.1, representing isolates with relevant CCNV variability. (*B*) Evaluation of CCNV in each chromosome in the population. Each dot corresponds to a different chromosome. The *x*- and *y*-axes represent, respectively, the CDO and CSV of each chromosome across each population. Chromosomes with CDO > 1.4 are identified by their numbers, representing chromosomes with consistent extra copies. The horizontal red dotted line represents the expected CDO value for euploid chromosomes. The horizontal and vertical blue dotted lines correspond, respectively, to CSV 0.1 and CDO 1.4. (*C*) Trypanosomatid maximum likelihood phylogeny, highlighting the clades that are aneuploid/mostly euploid. *Bodo saltans* (*Bsaltans*) was used as an outgroup to root the tree. Node numbers in the tree correspond to the percentage of bootstrap support. The color strip represents the presence/absence of aneuploidies in a given clade. Heatmaps representative of the CCNV for all isolates can be seen in Supplemental Figure S1.

We first evaluated presence and variability of CCNVs across isolates using two metrics: (1) isolate genome expansion (IGE), in which high values occur when several chromosomes are duplicated in a single isolate, and (2) isolate aneuploidy level (IAL), in which high values occurs when there is large intra-isolate variation in chromosome copies (Fig. 1A). We found clear evidence for consistent aneuploidies in isolates from all evaluated taxa, with the exception of three closely related monophyletic species: *T. brucei*, *Trypanosoma congolense*, and *Trypanosoma vivax*. Although 80%–100% of the *Crithidia*, *Endotrypanum*, *Leishmania*, *Leptomonas*, *Paratrypanosoma*, *Porcisia*, and *T. cruzi* isolates had an IAL higher than 0.10, only 0%–5% of the *T. brucei*, *T. congolense*, and *T. vivax* isolates were above this cutoff (Supplemental Table S4). The level of aneuploidy varied within clades, as we observed some isolates with high values of both IGE and IAL, such as *Leishmania* “ERR205781” (IGE 1.189 and IAL 0.311) and *Leptomonas* “SRR2087426” (IGE 1.31 and IAL 0.585), contrasting with others from the same clade that are mainly euploid: *Leishmania* “ERR4678144” (IGE 1.009 and IAL 0.071) and *Leptomonas* “SRR2045872” (IGE 1.011 and IAL 0.132) (Fig. 1A; Supplemental Table S4). The low IGE observed in most isolates (min = 0.912, mean = 1.02, max = 1.31) for all groups suggests that variations in chromosome copy number from the basal ploidy occur in just a few chromosomes in a given isolate. Our IGE estimations have some limitations, as they were performed using bulk DNA sequencing and using isolates from different culture conditions. In bulk sequencing, IGE values could be underestimated in balanced CIN variations, for example, if part of the population is trisomic and part is monosomic for the same chromosome, which could result in a somy estimation close to disomy. Similarly, chromosomal duplications observed in only a few cells would not be detected. Different media culture conditions and the number of culture passages could also impact IGE levels, as long-term culturing was shown to increase somy variation (Dumetz et al. 2017; Domagalska et al. 2019). Nevertheless, the restriction of overall chromosomal expansion at the populational level observed here may be caused by the high fitness cost of maintaining chromosomal-wide gene duplications, as seen in yeast (Pompei and Cosentino Lagomarsino 2023). Further representations of CCNV for all clades can be seen in Supplemental Figure S1.

It is well established that Chromosome 31 is consistently supernumerary in *Leishmania* species (Chromosome 30 in the *Leishmania mexicana* complex) (Britto et al. 1998; Rogers et al. 2011; Black et al. 2023). As aneuploidy is frequent in the majority of trypanosomatid clades, we went on to investigate if there were chromosomes that are consistently expanded in each clade. This analysis differs from the previous one as now we focused on chromosomes instead of isolates. Two metrics were used: (1) chromosome duplication occurrence (CDO), which represents chromosome mean copy number across isolates in the population for a particular chromosome, and (2) chromosome somy variation (CSV), which: represents how variable the presence of a given chromosomal copy gain/loss is across the population, for a particular chromosome (Fig. 1B; Supplemental Table S5). We defined “consistently supernumerary chromosomes” as the ones with an CDO higher or equal to 1.4 (close to at least trisomic in a diploid organism). We observed 16 consistently supernumerary chromosomes, comprising two in *Crithidia bombi* (0, 9), one in *Crithidia fasciculata* (27), one in *Endotrypanum* (31), one in *L. donovani* (31, as described previously) (Rogers et al. 2011), one in *Leishmania major* (31, as described previously) (Rogers et al. 2011), four in *Leptomonas* (12, 29, 32, and 39), three in *Paratrypanosoma* (36, 45, and 64), two in *Porcisia* (30526 and 30534), and one in *T. cruzi* (31 as described previously) (Reis-Cunha et al. 2015). The CDO of these chromosomes had values around 1.5 to 2, meaning 1.5 to two copies for each haploid genome copy. There were no chromosomes with a CDO higher or equal to 1.4 in *T. brucei*, *T. congolens*e, or *T. vivax*, consistent with our previous analysis suggesting aneuploidy is rare in these species. The gene CNV in these consistently supernumerary chromosomes had a coefficient of variation that was similar to or lower than the one observed for other chromosomes, which indicates that their increased copy number is caused by whole-chromosome duplication, rather than segmental duplication or CIN (Supplemental Fig. S2).

When the total evidence for presence/absence of aneuploidy was evaluated in context of a trypanosomatid phylogenetic tree (Fig. 1C), the presence of CCNV in distant clades (as in *T. cruzi*, *Leishmania*, and *Crithidia*), and in the two samples from the basal *Paratrypanosoma confusum* is a strong indicator that aneuploidy is an ancestral characteristic of trypanosomatids, which has later evolved to become largely absent in the monophyletic clade that encompasses *T. brucei* and closely related protozoans. In this scenario, the reorganization of *T. brucei* and related protozoan genomes into a reduced number of larger chromosomes (El-Sayed et al. 2005b) could increase the fitness cost of aneuploidies owing to the increased number of gene products with unbalanced proportions that are generated by each chromosomal duplication.

### Chromosomes that are consistently duplicated in other clades are syntenic to Leish Chr 31

**Figure 2.**
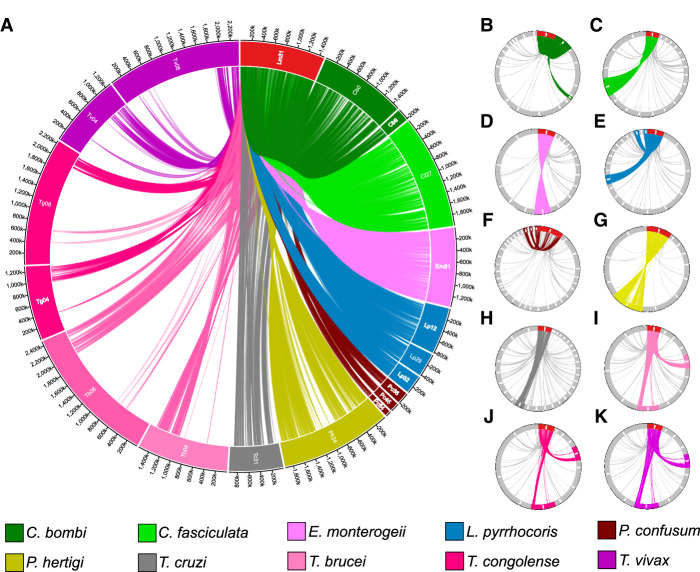
Chromosomes with a high density of orthologous genes with *Leishmania* Chromosome 31 (Leish Chr 31) are also present extra copies in other trypanosomatids. Circa plots representing the orthology between Leish Chr 31 (red box) and chromosomes from other species (colored boxes), drawn in proportion to their sizes. The presence of ortholog genes between Leish Chr 31 and a given chromosome is shown by linking lines. (*A*) Gene sharing between Leish Chr 31 and chromosomes that are consistently supernumerary in other clades (*TASC*) and in Chromosome 4 and 8 from *T. bruce*i and closely related clades. (*B*–*K*) All orthologs between Leish Chr 31 genes and genes in any chromosome from other clades, separated by species. Only chromosomes that share at least one ortholog gene with Leish Chr 31 are shown. Chromosomes that are consistently supernumerary are highlighted by colors, whereas other chromosomes are in light gray. (*B*) *Crithidia bombi*; (*C*) *Crithidia fasciculata*; (*D*) *Endotrypanum*; (*E*) *Leptomonas*; (*F*) *Paratrypanosoma*; (*G*) *Porcisia*; (*H*) *Trypanosoma cruzi*; (*I*) *Trypanosoma brucei*; (*J*) *Trypanosoma congolense*; (*K*) *Trypanosoma vivax*. Larger versions of these plots can be seen in the Supplemental Figure S4.

Chromosome 31 (Chromosome 30 in the *L. mexicana* complex) is consistently expanded in all *Leishmania* species and isolates evaluated to date (Rogers et al. 2011; Mannaert et al. 2012; Black et al. 2023). We observed that the majority of consistently expanded chromosomes in other clades shared many orthologs with *L. major* Chr 31, varying from 51% in *T*. *cruzi* to 93% in *L. donovani* (Fig. 2A–K; Supplemental Fig. S3). Even the typically euploid *T. brucei* and *T. vivax* had Leish Chr 31 genes with orthologs simultaneously in two chromosomes, Chr 4 and Chr 8, as described previously (Jackson 2007; Marques et al. 2015). The widespread sharing of orthologous genes suggests that the expansion of Leish Chr 31 is ancient, predating the separation of the trypanosomatid clade and the genome reorganization seen in *T. brucei* and persisting across this extensive evolutionary history. Because of this finding, we went on to evaluate the gene structure and variability of this chromosome, which will be collectively called the *trypanosomatid ancestral supernumerary chromosome* (*TASC*).

### *TASC* has a peculiar gene structure, an increased nucleotide diversity, and minor allele frequency

**Figure 3.**
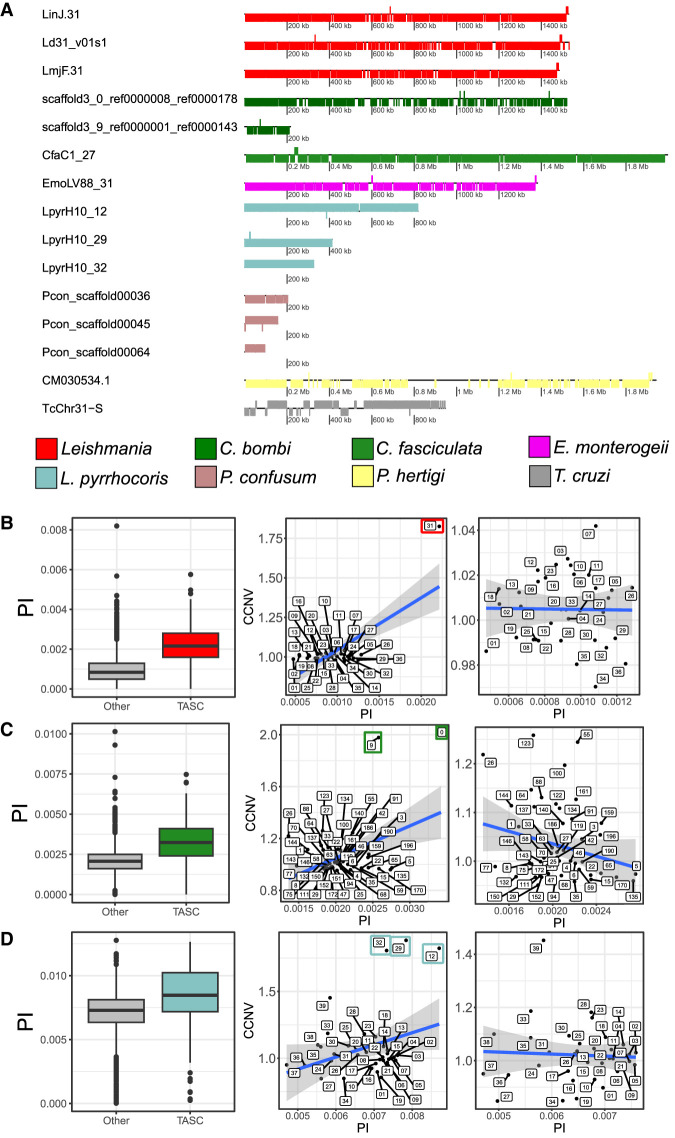
*TASC* has almost all genes in the same coding strand and an increased nucleotide diversity. (*A*) Gene distribution of all *TASC*. Each box corresponds to a gene, and the position of the box *below* or *above* the strand corresponds to the gene orientation. The colors represent the species/clades of origin of each chromosome. All the genes are usually in the same coding strand. Nucleotide diversity and comparison between diversity and CCNV for the *L. donovani* EA_HIV set (*B*), *C. bombi* (*C*), and *Leptomonas* (*D*). *Left* panels are box plots representing 10-kb window π-values comparing *TASC* and other chromosomes. Chromosomes from *TASC* are colored, whereas others are gray. (*Middle*) Correlation between CCNV (*y*-axis) and π (*x*-axis) in all chromosomes. (*Right*) Without *TASC*. The *TASC* are highlighted by colored boxes. *L. donovani* EA_HIV set: (*middle*) *r* = 0.719, *P*-value 7.58 × 10^−7^; (*right*) *r* = −0.001, *P*-value = 0.9943. *C. bombi*: (*middle*) *r* = 0.429, *P*-value 0.001082, (*right*) r = −0.229, *P*-value = 0.09815. *Leptomonas*: (*middle*) *r* = 0.309, *P*-value 0.05542; (*right*) *r* = −0.133, *P*-value = 0.4392.

We first evaluated structural and functional characteristics of *TASC*, including gene content, nucleotide diversity, and minor allele frequency (MAF). Of the 352 genes annotated in *L. major* Chr 31, 349 (99.1%) are in the same coding strand (Fig. 3A). Similar proportions can be observed in other species, varying from 87.3% in *T. cruzi* to 99.4% in *Leptomonas*, which is unusual for large chromosomes (Fig. 3A; Supplemental Fig. S5). Strand switch regions (SSRs) are important in trypanosomatids, in which some act as replication origins and as the transcription start and termination sites for polycistrons (Siegel et al. 2009; Tiengwe et al. 2012; Marques and McCulloch 2018; Kraus et al. 2020). Thus, having almost all genes on the same strand could impact DNA replication and/or gene expression.

One anticipated consequence of maintaining a duplicated chromosome long-term is a higher number of segregating sites (genome positions that vary in the population) (Supplemental Fig. S6) and nucleotide diversity (π) (Supplemental Fig. S7). The number of segregation sites (Supplemental Table S6) and nucleotide diversity were estimated in the three aneuploid lineages with more than 10 isolates: *L. donovani*, *C. bombi*, and *Leptomonas* (Fig. 3B–D). *T. cruzi* was not evaluated, as the expansion of multigene repetitive family clusters in this clade could compromise genome-wide diversity estimations. As expected, there was a higher nucleotide diversity in *TASC* for the three clades compared with the combination of all other chromosomes, using 10-kb sliding windows (*L. donovani P-*value = 1.5 × 10^−47^; *C. bombi P*-value 3.47 × 10^−39^; *Leptomonas P*-value 1.08 × 10^−12^), suggesting that the observed higher diversity in these chromosomes was not the result of a few highly polymorphic regions (Fig. 3B–D; Supplemental Fig. S7). We observed a significant positive correlation between the population mean chromosomal copy number for each chromosome and its nucleotide diversity, when all chromosomes were evaluated (Fig. 3B–D). However, this correlation was lost when the *TASC* was removed, suggesting that ploidy variation for the other chromosomes is transient and not maintained long enough to impact their overall nucleotide diversity.

We also observed some genes with a higher nucleotide diversity than expected in *TASC* for the three clades (higher than the mean + 3 SD from the genomic mean π), in which the majority were hypothetical proteins or pseudogenes (Supplemental Table S7). Pseudogenes are usually under no or weak purifying selection, which explains their increased sequence diversity.

We next went on to evaluate the biological impact of the segregating sites and MAF in *TASC* using *L. donovani*, as it has the most accurate genome assembly and annotation from the three clades *L. donovani*, *C. bombi*, and *Leptomonas*. Leish Chr 31 had an increase of SNP counts per kilobase, which was more prominent in intergenic regions (1.8×) than in synonymous (1.41×) and nonsynonymous (1.58×) sites, compared with other chromosomes (Supplemental Fig. S6). This is expected, as missense SNPs are usually more deleterious than intergenic mutations and should accumulate at a lower rate in the population. Both *TASC* and other chromosomes have a MAF peak at low values, but *TASC* had a higher tail toward 0.5 MAF (Supplemental Fig. S6). This finding might suggest that there are more balanced alleles in Chr 31, which could be an indication of sites under balancing selection in this chromosome (Grace et al. 2021). Nucleotide diversity and MAF estimations were not greatly impacted by using diploid or tetraploid SNP callers (Supplemental Fig. S6).

### TASC has increased potential for functional variation but purge highly deleterious mutations

As TASC consistently has extra copies and a higher nucleotide diversity compared with other chromosomes, we evaluated if this could impact the tolerance to deleterious mutations, using population data from 113 *L. donovani* isolates from East Africa. We initially evaluated high-impact variations, such as stop-codon gain in protein-coding genes and stop-codon loss in pseudogenes. We only observed seven of these variations in TASC, which were not informative: redundant stop codons in pseudogenes, observed in only one isolate, or did not impact the gene function (Supplemental Fig. S8).

**Figure 4.**
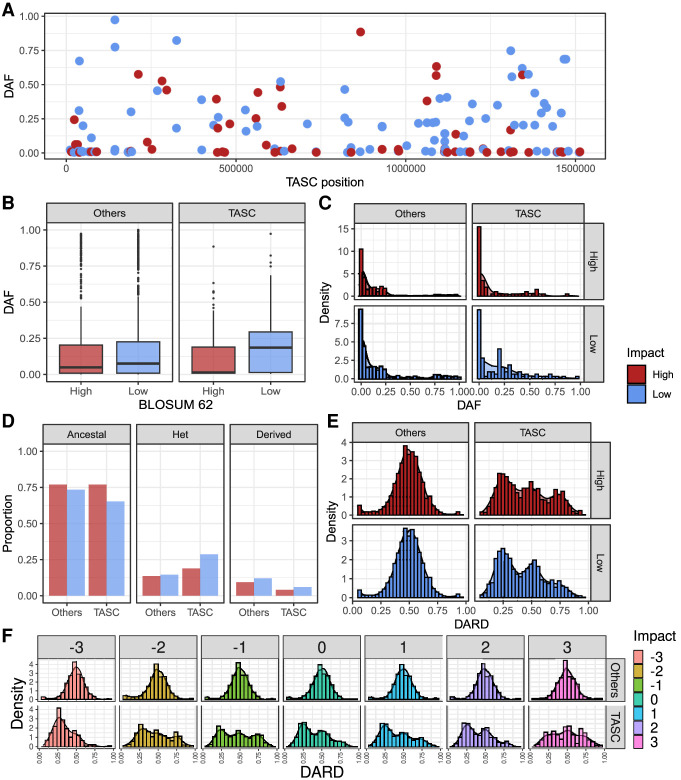
TASC carry more potential for increased functional variation but purge highly deleterious mutations more efficiently than other chromosomes. (*A*) SNP-derived allele frequency (DAF) along TASC. Each dot corresponds to a SNP; the *y*-axis to its DAF and the *x*-axis to Leish Chr 31 positions. (*B*,*C*) Boxplot (*B*) and density plot (*C*) comparing DAF from TASC and other chromosomes. (*D*) Proportion of ancestral homozygous (ancestral), heterozygous (Het), or derived homozygous (derived) genotypes in the 113 *L. donovani* isolates. Distribution of derived allele read depth (DARD) in heterozygous positions on TASC and other chromosomes, separated by high and low impact (*E*) or by −3 to 3 impact (*F*).

As the high-impact SNPs were noninformative, we classified the potential impact and ancestrality of nonsynonymous SNPs in TASC. To that end, we first identified the potential ancestral allele based on conservation of the amino acid position among the protein sequences in the trypanosomatid species. Then, we determined the impact of each mutation based on its amino acid BLOSUM 62 substitution score, in which negative values correspond to uncommon and potentially highly impactful amino acid replacements, and positive values correspond to potentially less impactful amino acid changes. We obtained a total of 1829 nonsynonymous segregating sites for this analysis, in which 156 were in TASC, spread along the chromosome (Fig. 4A).

First, to check if potentially deleterious (derived) variations were kept in low frequency in the population, we evaluated the derived allele frequency (DAF; proportion of alleles that correspond to the derived allele in the population). There was a lower DAF for high-impact modifications (BLOSUM 62 score less than zero) compared with low-impact modifications (BLOSUM 62 score equal to or greater than zero), both for TASC (Mann–Whitney *U* test *P*-value = 0.0026) and other chromosomes (Mann–Whitney *U* test *P*-value = 0.0117). This suggests that deleterious mutations are often removed from all chromosomes more efficiently than low-impact mutations, as expected under purifying selection (Fig. 4B,C). TASC had a lower DAF for high-impact SNPs (Mann–Whitney *U* test *P*-value 0.0420) and a higher DAF for low-impact SNPs (Mann–Whitney *U* test *P*-value 0.0525) compared with other chromosomes, consistent with stronger purifying selection acting on the TASC than elsewhere in the genome. This observation is consistent with the expectation from population genetics that deleterious mutations will be removed more rapidly in a population in an increased effective population size (Lanfear et al. 2014). In this case, TASC maintains twice the effective population size of the other chromosomes. The higher DAF for low-impact SNPs in TASC was caused by an increase in the proportion of isolates with heterozygous sites and a reduction of ancestral homozygous genotypes compared with other chromosomes. On the other hand, the lower DAF for high-impact SNPs in TASC was mainly caused by a reduction in the number of homozygous for the derived allele (Fig. 4D).

When we evaluated only the heterozygous sites for each segregating site position of each of the 113 *L. donovani* isolates, using the derived allele read depth (DARD), there were more SNPs with three copies of the ancestral allele than with three copies of the derived allele (Fig. 4E). These could be recently acquired mutations or a potential selection to maintain deleterious SNPs in only one or two of the four copies. When we separated the DARD distributions by BLOSUM 62 impact, the most potentially deleterious mutations (impact −3) had a lower DARD than the least deleterious mutations (impact 3) (Mann–Whitney *U* test *P*-value 0.0125) (Fig. 4F). Taken together, these data suggest that TASC may be purging highly deleterious SNPs from the population more efficiently while maintaining low-impactful SNPs in higher proportions than other chromosomes and maintaining the alleles in heterozygous sites in one or two of the four chromosome copies.

### Gene expression from Leish Chr 31 is controlled by down-regulation of all chromosomal copies to a similar extent

Previous works from Prieto Barja et al. (2017) and Dumetz et al. (2017) have demonstrated that having extra chromosomal copies in *Leishmania* results in a proportional increase in overall gene expression. The only exception to these observations is Leish Chr 31, which has a gene expression similar to disomic chromosomes despite having three to four copies. How gene expression control is achieved on Leish Chr 31 is unknown. We propose two main hypotheses for this regulation: (1) *Leishmania* down-regulates the expression of all the approximately four copies of Chr 31 to a similar extent, and (2) *Leishmania* silences two whole-chromosome copies, leaving two copies normally expressed. To investigate this, we used seven available pairs of WGS and RNA-seq data described by Prieto Barja et al. (2017) and estimated the variation in the proportion of reads in heterozygous positions in both data sets (see methods).

**Figure 5.**
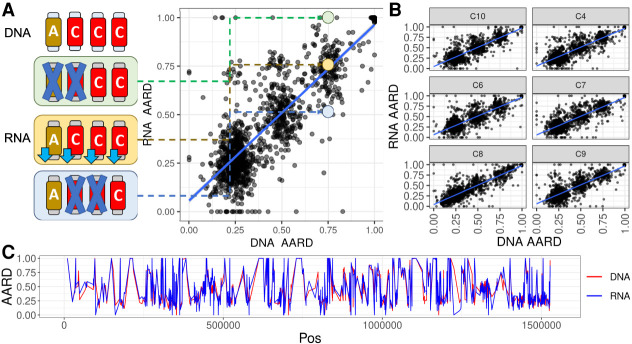
Leish Chr 31 gene expression is similar to disomic chromosomes, and the four copies appear to be down-regulated similarly. (*A*) Correlation between DNA AARD (*x*-axis) and RNA AARD (*y*-axis) for each SNP position in clone 3 (C3) from the tetraploid Leish Chr 31. Each dot represents one SNP position from the Leish Chr 31 in the C3 (*r* = 0.872, *P*-value = 0). On the *left*, a scenario in which four alleles are “A,” “C,” “C,” and “C,” and “C” is the alternate allele; therefore, WGS AARD = 0.75%. If the gene expression of all chromosome copies is silenced similarly, the AARD from the RNA-seq data for the same position would also be around 0.75 (yellow box and dot). If two chromosomal copies are silenced, this would result in “AC” with an AARD of 0.5 or “CC” with an ARDD of 1.0 (blue and green boxes and dots, respectively). (*B*) AARD DNA–RNA correlation plots for the Leish Chr 31 in clones C10 (*r* = 0.887, *P*-value = 0), C4 (*r* = 0.875, *P*-value = 0), C6 (*r* = 0.868, *P*-value = 0), C7 (*r* = 0.869, *P*-value = 0), C8 (*r* = 0.905, *P*-value = 0), and C9 (*r* = 0.866, *P*-value = 0). (*C*) DNA (red line) and RNA (blue line) AARD along Leish Chr 31 in C3. The other six clones can be seen in Supplemental Figure S9.

We initially confirmed the results from Prieto Barja and colleagues (2017), confirming that the expression profile of Chr 31 is similar to a disomic chromosome despite its supernumerary nature (Supplemental Fig. S9). Using the same data set, we evaluated if the alternate allele read depth (AARD) in heterozygous positions from the WGS data correlates with the AARD from the RNA-seq data (Fig. 5A,B; Supplemental Table S8). A strong positive correlation (*r* ∼ 0.88) between paired WGS and RNA-seq AARDs from seven *L. donovani* clones was observed, strongly suggesting that all copies are being silenced similarly. This conclusion is supported by evaluation of AARD variation along the chromosome, which shows a clear match between the WGS and RNA-seq data (Fig. 5C; Supplemental Fig. S9). This regulation does not appear to be caused by codon usage, as there was no significant difference among *Leishmania* chromosomes (Supplemental Fig. S10).

### Biological functions of genes and regions from TASC

We next evaluated potential biological functions for genes in *TASC* to assess if there is any enriched function within this constantly expanded chromosome. This analysis was performed using *L. donovani* Chr 31, *T. cruzi* Chromosome 31, and *T. brucei* regions from Chromosome 4 and Chromosome 8 that are syntenic to *TASC*, as these genomes have the most curated annotations from the evaluated species. We separated the data set in three main groups: (1) conserved_all, genes that were present in orthogroups containing the three clades; (2) conserved_pair, genes in orthogroups containing only two clades; and (3) exclusive, genes that were exclusive for each clade. We compared the Gene Ontology (GO) from these data sets with a data set containing all genes for the three species.

When the genes from the conserved_all data set were evaluated, the corresponding enriched biological functions were associated with basal cellular metabolism, (Supplemental Table S9). For group 2 conserved_pair, we found an enrichment of GO terms related to amino-acid transport for the pair *T. cruzi* and *L. donovani*, which might be important for their intracellular survival. But the most relevant enrichment were GO terms associated with the “glycosylphosphatidylinositol (GPI) anchor biosynthetic process” (∼25% of the genes), “protein glycosylation” (∼25% of the genes), and “UDP-glycosyltransferase activity” (∼43% of the genes) obtained for the pair *T. brucei* and *T. cruzi*. The GPI anchor is a crucial structure in these protozoans, used to anchor proteins to the outer membrane, including virulence factors, like the variant surface glycoproteins (VSGs) from *T. brucei,* and, from *T. cruzi*, mucin (TcMUC), mucin-associated surface proteins (MASPs), and *trans*-sialidases (TSs). Thus, genes in *TASC* could play important roles in GPI assembly and glycosylation in these protozoans. There was no relevant enriched function detected between the *T. brucei–L. major* pair.

Finally, for the group 3, exclusive, there were only a few functional enrichments, such as “phosphatidylcholine biosynthetic process” for *L. major*, “cAMP biosynthetic process” for *T. brucei*, and “arginine biosynthetic process” and “deacetylase activity” for *T. cruzi* (Supplemental Table S9). It is worth mentioning that some important drug-resistance targets in *Leishmania* are also found on this chromosome, including the miltefosine susceptibility locus (MSL) (Carnielli et al. 2018) and the aquaglyceroporin gene (Potvin et al. 2021), in which the resistance are caused by segmental deletions or gene inactivation.

Taken together, these results suggest that the genes in *TASC* that were shared among species are involved in cellular basal metabolism and that some species have transferred genes related to their specific parasitic lifestyle to this chromosome.

## Discussion

By evaluating chromosome ploidy across genera that span the trypanosomatid grouping, we have revealed three features of aneuploidy in these parasites. First, aneuploidy tolerance is an ancestral characteristic of trypanosomatids, suggesting it is central to their genome functionality. Second, *T. brucei* and related African trypanosomes have more recently evolved to largely dispense with aneuploidy, perhaps reflecting genome reorganization. Third, we have identified the presence of an ancestral chromosomal duplication, named collectively as “*TASC*,” which has been maintained throughout trypanosomatid evolution either as a greater than diploid chromosome or as a syntenic duplication in two chromosomes in African trypanosomes, resulting in higher nucleotide diversity, unusual gene organization, gene expression regulation, and evolution. This is the most comprehensive evaluation of aneuploidies across trypanosomatids to date.

Previous works have described aneuploidy in distant trypanosomatid clades, mainly represented by several *Leishmania* species (Rogers et al. 2011), and *T. cruzi* DTUs (Reis-Cunha et al. 2015, 2018). Taking advantage of near complete genomes of clades across the trypanosomatids, we reveal that aneuploidy is widespread throughout these protozoans. Isolate-specific data indicate that the overall level and variation in chromosomal expansions are low, which suggest that aneuploidy is normally limited to only a few chromosomes in the same isolate. This suggests that the increased fitness cost that they incur usually outweighs the fitness gain of having specific genes in extra copies (Pompei and Cosentino Lagomarsino 2023). *T. brucei* and closely related clades are unusual among trypanosomatids by being mostly euploid, which suggests that the cost of aneuploidy could be higher in these species (discussed further below) (Tihon et al. 2017a,b; Almeida et al. 2018; Cosentino et al. 2021). The widespread presence of aneuploidies across distantly related clades, as well as the restricted but not absent occurrence of aneuploidy in *T. brucei*, strongly supports that aneuploidy is an ancestral characteristic suppressed in *T. brucei* and closely related protozoans. More isolates from the basal clade *P. confusum* and more distant protozoan clades as the *Bodo saltans* could help in elucidating where the tolerance to aneuploidies arose.

The strong reduction of aneuploidies in *T. brucei* and closely related parasite clades suggests that not only the environment but specific genomic modifications in this group may explain why chromosomal duplications are more restricted. Some of these modifications are: megabase-sized chromosomes, minichromosomes, and differences in DNA replication regulation. *T. brucei* and closely related protozoans have their genome organized into 11 large megabase-size chromosomes, whereas *T. cruzi* and *Leishmania* have about 41–47 and 34–36 shorter chromosomes, respectively (Berriman et al. 2005; El-Sayed et al. 2005a,b; Peacock et al. 2007; Weatherly et al. 2009; Jackson et al. 2010, 2012; Wang et al. 2021). Hence, the presence of one chromosome with extra copies in *T. brucei* might, on average, amplify more genes than one in *T. cruzi* or *Leishmania*. In this scenario, the fitness cost of having unwanted genes amplified could outweigh the benefits of having just few beneficial genes with extra copies, as proposed in yeast (Pompei and Cosentino Lagomarsino 2023). Why *T. brucei* and close relatives might have evolved a smaller repertoire of larger chromosomes is unclear, but as part of the reorganization, parts of *TASC* were maintained in increased copy number through duplication in *T. brucei* Chromosomes 4 and 8. Another mitigating factor for the lack of aneuploidies in *T. brucei* might be owing to the presence of “*minichromosomes*,” which vary in size between 30–150 kb, with an estimated approximately 100 copies per cell (Wickstead et al. 2004). These short chromosomes usually contain one or two genes coding for VSG, which is important in the antigenic variation process in these parasites (for review, see Glover et al. 2013). These minichromosomes could potentially vary in copy and content among *T. brucei* cells, mitigating the need for whole-megabase chromosomal aneuploidies. However, to date, only VSG genes and VSG transcription sites have been found to be present in minichromosomes, so how these features could contribute to adaptive gene expression is unclear. Finally, there are several differences between DNA replication in *T. brucei* and *Leishmania* (for review, see Damasceno et al. 2021). MFA-seq experiments have shown that although *T. brucei* possesses multiple replication origins per chromosome, *Leishmania* appears to initiate DNA replication from a singular site on each chromosomes during the S phase, which is less than the predicted number of origins required to complete replication of the larger chromosomes (Marques et al. 2015; Damasceno et al. 2020, 2021). To circumvent this problem, *Leishmania* appears to confine duplication of the core genome to S phase with duplication of the subtelomeres occurring later, during late S, G_2_/M, and G_1_ stages of the cycle (Damasceno et al. 2020). Replication outside of S phase is common in aneuploid cells, including aneuploid cancers (Minocherhomji et al. 2015; Özer et al. 2018). Short nascent DNA strand sequencing (SNS-seq) suggests the presence of numerous additional and alternative origins of replication in *Leishmania* (Lombraña et al. 2016), potentially reflecting origins differently activated within a cell population (Damasceno et al. 2021). This might result in a diversity of replication programs and, potentially, mosaic aneuploidy, which could explain the within clone variation described by Negreira et al. (2022). Hence, the differences in the susceptibility to aneuploidy could be directly related to genome organization and differences in replication processes.

By evaluating chromosomal duplications, we have shown that the majority of consistently supernumerary chromosomes in all evaluated species are syntenic to Leish Chr 31, now collectively called *TASC*. Even though the presence of extra copies of Chromosome 31 and the synteny with regions from *T. brucei* were previously reported (Jackson 2007; Rogers et al. 2011; Marques et al. 2015; Prieto Barja et al. 2017), our data reveal for the first time that this stable increase in ploidy is a universal feature of trypanosomatids, predating the speciation of the evaluated clades, and has been maintained across their evolution. We found no other example of a stable increase in ploidy for any other chromosome across any of the trypanosomatids we examined herein, which suggests that the presence of extra copies from other chromosomes is transient and potentially context dependent.

One striking feature of the consistent tetraploidy shown by *TASC* is that it appears to have impacted upon gene structure, expression control, and evolution. Although consistently present in additional copies, Leish Chr 31 is usually expressed as if it were disomic (Dumetz et al. 2017; Prieto Barja et al. 2017). As this chromosomal duplication is ancient, these parasites might have evolved a mechanism to regulate its gene expression, potentially counteracting the deleterious effects of a constant imbalance in gene copy number arising from this chromosome. This expression control could be generated by fully silencing chromosomal copies, akin to the silencing of the human X Chromosome mediated by the ncRNA *XIST* and irreversible heterochromatization (Lee 2011; Lee and Bartolomei 2013; Petropoulos et al. 2016), or, by down-regulating to a similar level, different chromosomal copies via an unknown mechanism. By comparing the AARD in heterozygous positions in WGS and RNA-seq along Leish Chr 31 in clones (data from Prieto Barja et al. 2017), we have shown that, at least at the population level, *L. donovani* potentially regulates the gene expression by similarly down-regulating all chromosome copies and that this appears to be independent of codon usage variation (Zhou et al. 2016). With the current data, we cannot exclude the possibility that, at least in vitro, the silencing of Chr 31 copies may vary at the level of the individual, resulting in a globally similar level of expression for all chromosomal copies. If that is the case, the rate in which the chromosome silencing changes in *L. donovani* would have to be high, as the analysis was performed using clonal isolates. Single-cell RNA-seq data will be the key to elucidating the haplotype-specific expression of Leish Chr 31. Another peculiarity of Leish Chr 31 (and in all *TASC*) that could impact gene expression is the arrangement of the majority of genes on the same coding strand. SSRs are important genomic regions in trypanosomatids, because they define the ends of polycistronic units, playing roles in transcription initiation and termination, as well as in being sites of DNA replication initiation (Tiengwe et al. 2012; Marques et al. 2015; Marques and McCulloch 2018). Hence, having almost all genes on the same coding strand could impact gene expression regulation of this chromosome. Future studies with the careful evaluation of gene expression mechanisms in this chromosome might help in elucidating if this control occurs at the transcription level.

From another perspective, the evolutionary pressure to keep extra copies of Leish Chr 31 might be independent of gene expression regulation and, instead, relate to increasing sequence variability potentially promoting neofunctionalization of paralogous genes, as proposed by Jackson (2007) pertaining to the duplication in *T. brucei* Chromosomes 4 and 8. In this scenario, having extra copies could allow mutations to accumulate differentially in each haplotype, and more suitable variants could be expanded by haplotype selection (Prieto Barja et al. 2017). This is in agreement with both the higher nucleotide diversity (π) and the higher tolerance to low-impactful mutations that we observed in this chromosome. The consistent polysomy of this chromosome allows the presence of low-impactful SNPs in higher proportions in the population, especially in heterozygous positions with one or two copies, which could be explored by the parasite to generate controlled sequence variability. On the other hand, Leish Chr 31 purges highly deleterious SNPs more efficiently compared with other chromosomes, which is in accordance with what is expected for larger effective population sizes (Ne) (Lanfear et al. 2014).

Finally, we evaluated the biological functions that were enriched in the shared core, or in species-specific regions from *TASC*, focusing on the “TriTryps” *Leishmania* (Chr 31), *T. brucei* (Chromosome 4 1 Mb:1.5 Mb and Chromosome 8 2 Mb:2.5 Mb), and *T. cruzi* (Chromosome 31). The majority of biological functions enriched in the shared content were related to basal cellular processes. Notably, functions that were enriched solely in *T. brucei* and *T. cruzi* were linked to glycosylation and surface protein anchoring. GPI-anchored proteins play a pivotal role in host–pathogen interactions, including immune evasion and host cell invasion processes (Glover et al. 2013; Horn 2014). UDP-glycosyltransferases catalyze the transfer of *N*-acetylglucosamine residues from UDP-GlcNAc to phosphatidylinositol, which is crucial not only in the initial steps of GPI anchor synthesis but also in the synthesis of several glycans in the surface of trypanosomatids (Doering et al. 1989; Güther et al. 2006; Silva Pereira and Jackson 2018). In *T. cruzi*, the proteins encoded by the multigene families MASP, mucin, and *trans-*sialidase, which act in cellular adhesion, invasion, and immune evasion processes (for review, see De Pablos and Osuna 2012), are anchored to the surface of the parasite by GPI-anchors, where mucins are also highly glycosylated (Buscaglia et al. 2006). In *T. brucei*, VSGs, the proteins involved in immune evasion via antigenic variation, are also anchored to the parasite's surface by GPI anchors (Ferguson et al. 1988; Horn 2014). Although not significantly enriched by GO term analysis in *Leishmania*, several drug-resistance genes or regions are found on Leish Chr 31, including the MSL (Carnielli et al. 2018) and the aquaglyceroporin gene (Potvin et al. 2021), and are both a result of gene inactivation or deletion. It is tempting to speculate that chromosomes with stable extra copies might be more prone to gene inactivation without loss of function. In this scenario, the loss of function of some genes on one or two chromosome copies could be compensated, at least partially, by the functional genes found on the other copies under natural conditions. Under drug pressure or in the presence of other stressors, if deleting the region(s) is advantageous, such alterations to the chromosome may be selected upon and even expanded by mechanisms such as haplotype selection (Prieto Barja et al. 2017). In fact, if these deletions and/or frameshifts reduce fitness, they may exist in lower dosages (i.e., one of the four copies) in some isolates, mitigating their detrimental effects in the absence of stress and increasing in number if required. Aneuploidies are commonly associated with drug-resistance emergence in cancer, in which resistant cells harbor recurrent aneuploidies (Lukow et al. 2021), and in yeast (Selmecki et al. 2006, 2009). Taken together, these results suggest that the expansion of *TASC* has impacted upon shared housekeeping genes and also host–parasite interaction and drug-resistance genes/loci.

Herein, we have shown that aneuploidy is an ancestral characteristic of the trypanosomatid clade and that TASC have higher nucleotide diversity, unusual gene organization, novel expression regulation, and evolution compared with other chromosomes. What processes govern aneuploidy in these protozoans and the underlying molecular mechanisms that regulate TASC expression remain unknown. New studies investigating these modifications will be important to address these deficits in our understanding. Moreover, a complete genome assembly of the outgroup protozoan *B. saltans* may shed light on persistence of aneuploidy and TASC expansion across the wider kinetoplastid grouping.

## Methods

### Reference genomes, WGS read library preprocessing, mapping, and chromosomal somy estimation

All data sets used in the study were obtained from public repositories: NCBI SRA (https://www.ncbi.nlm.nih.gov/sra) and TriTrypDB (https://tritrypdb.org/tritrypdb/app). The sample and genome IDs can be obtained in the Supplemental Tables S1, S2, and S3.

To estimate the occurrence of aneuploidies in Trypanosomatidae, we evaluated aneuploidy presence across seven different genera, using 866 WGS Illumina sequencing data sets (Supplemental Tables S1, S2). Read mapping and coverage estimation were performed using standard protocols (Supplemental Methods).

The intra-isolate assessment of aneuploidy and chromosomal duplication was measured using two metrics: the isolate mean chromosome copy number, representing within-IGE, and the isolate standard deviation of chromosomal copies, which represents within-IAL. For IGE, the somy of each chromosome in an isolate is summed and divided by the number of chromosomes. The IAL corresponds to the standard deviation of chromosomal copies within an isolate. The population variation of each specific chromosome copy was also measured with two metrics: chromosome mean copy number across isolates, representing CDO, and chromosome copy standard deviation across isolates, which represents CSV. For CDO, the mean copy number of a given chromosome was estimated by summing the values for the same chromosome in each isolate and dividing by the number of isolates, whereas CSV represents the standard deviation of the copy of the same chromosome across isolates. All estimations were scaled by haploid genome copies, in which a value of one corresponds to one copy for each haploid genome copy. Mean and standard deviation of chromosomal copies within isolates and within chromosomes were estimated using R (v.4.2.2) (R Core Team 2022).

### Phylogenetic and gene synteny analysis

The trypanosomatids phylogeny was estimated by maximum likelihood using IQ-TREE 2 v2.2.0 (Minh et al. 2020), with the single copy genes that were shared in 12 different Kinetoplastida groups defined by OrthoFinder (Supplemental Table S3). The full methodology is described in the Supplemental Methods.

### Gene sharing and gene organization in Leish Chr 31 and related sequences

The orthologous genes between *L. major* Chr 31 and chromosomes/scaffolds in each of the other species/groups listed in Supplemental Table S1 were identified using OrthoFinder v.2.5.4 (Emms and Kelly 2015, 2019). The chromosomes/scaffolds with 10 or more shared ortholog genes with Leish Chr 31 were identified. Chromosomes/scaffolds with fewer than 10 shared ortholog genes were grouped in the “others” data set. The bar charts representing the number of ortholog genes in each chromosome/scaffold for each species/group were generated in R (v.4.2.2) (R Core Team 2022). The gene coordinates and orientation of each ortholog gene, as well as the chromosome/scaffold size, were recovered from the general file format (GFF) files, obtained from NCBI, TriTrypDB v55, or as supplemental data in the work of Schmid-Hempel et al. (2018), in accordance with Supplemental Table S1. The synteny circular plots were generated with Circa (https://omgenomics.com/circa/). The chromosomal gene disposition plots were generated in R (v.4.2.2) (R Core Team 2022) using the library genoPlotR (Guy et al. 2010).

### Nucleotide diversity estimation

The nucleotide diversity (π) was estimated in three representative data sets that contained more than 10 isolates and evidence of consistent aneuploidy—*L. donovani* EA_HIV set (113 samples), *C. bombi* (29 samples), and *Leptomonas* (16 samples)—using VCFtools V.0.1.16 (Danecek et al. 2011) and was corrected to represent all windows in R (v.4.2.2) (R Core Team 2022). *T. cruzi* was not used owing to its large content of repetitive multigene families (El-Sayed et al. 2005a; Berná et al. 2018; Wang et al. 2021). Read filtering, mapping, SNP calling, and diversity estimation approaches were performed with standard tools (see Supplemental Methods).

### Gene expression control in *Leishmania* Chr 31

To evaluate the gene expression regulation in *L. donovani* Chr 31, a data set containing six parasite clones with matched WGS and RNA-seq previously described (Supplemental Table S8; Prieto Barja et al. 2017) was used. Both the WGS and RNA-seq read libraries were downloaded, polished, and mapped to the *L. donovani* LV9 reference genome as described in the Supplemental Methods. To assess if the overall gene expression in a chromosome was impacted by its copy number (confirming the results shown by Prieto Barja et al. 2017), the chromosomal coverage obtained using the WGS reads and the RNA-seq reads was estimated with SAMtools v.1.10 Coverage (Li et al. 2009) and normalized by the genome coverage, estimated as the mean coverage of all chromosomes. The correlation between the chromosome copy number and expression was estimated using the cor.test function in R (v.4.2.2) (R Core Team 2022).

Next, to assess how *L. donovani* regulates the gene expression of Leish Chr 31, we compared the AARD in heterozygous positions in both WGS and RNA-seq libraries in the same clone. There are two main scenarios: (1) all Leish Chr 31 chromosomal copies are down-regulated similarly, and (2) two chromosomal copies are silenced and two are expressed. In unbalanced heterozygous positions, as in a SNP position in a tetrasomic chromosome with three chromosomes with an alternate allele “C” and one chromosome with a reference allele “A,” resulting in “ACCC,” the WGS AARD would be 0.75. In the scenario 1, the expression of the four chromosome copies would be silenced similarly, causing the RNA-seq AARD to match the WGS AARD (i.e., 0.75). On the other hand, in scenario 2, if chromosomes “AC” were expressed and “CC” were silenced, the AARD would be lower (0.5), or if chromosomes “AC” were silenced and “CC” were expressed, it would be higher (1.0). Hence, a positive correlation between WGS and RNA-seq in heterozygous SNP regions would be an indication of the scenario 1, whereas a lack of correlation would be indicative of scenario 2. The SNP call and filtering were performed as described in the Supplemental Methods. To estimate the AARD, the multisample VCF was imported in R (v.4.2.2) (R Core Team 2022), converted in a table using the library vcfR (Knaus and Grünwald 2017), and processed with custom R scripts (see Software availability). The correlation between the AARD in the WGS and RNA-seq data sets was estimated using cor.test, in R (v.4.2.2) (R Core Team 2022).

To evaluate if the lower expression in Leish Chr 31 could be caused by a differential codon usage, the measure independent of length and composition (MILC) value was estimated for each chromosome, using the R library coRdon (https://bioconductor.org/packages/release/bioc/html/coRdon.html) and the *L. donovan*i LV9 annotated CDS downloaded from TriTrypDB v55. The script for this analysis is available at GitHub (see Software availability).

### Comparison of disomic or tetrasomic SNP callers in Chr 31 MAF, nucleotide diversity, and synonymous/nonsynonymous mutation estimations

To evaluate the impact of somy aware calling SNPs in Leish Chr 31, we performed the SNP calling in Leish Chr 31 as described in Supplemental Methods, using the *L. donovani* EA_HIV data set and ploidy values of two or four. The nucleotide diversity and MAF were estimated using custom scripts in R (v.4.2.2) (R Core Team 2022) optimized to disomic or tetrasomic data (see Software availability). The number, location, and potential biological impact of synonymous and nonsynonymous SNPs was estimated with SnpEff v.5.0d (Cingolani et al. 2012), and the data were summarized in R (v.4.2.2; R Core Team 2022).

### Evaluation of DAF and DARD, and the potential biological impact of nonsynonymous SNPs in *L. donovani* chromosomes

The segregating sites from the *L. donovani* East Africa population (113 isolates) (Supplemental Table S2) were classified as synonymous, nonsynonymous, start codon loss, stop codon gain, and stop codon loss with SnpEff v.5.0d (Cingolani et al. 2012). To identify derived and ancestral alleles, protein sequences from each orthogroup generated by OrthoFinder v.2.5.4 (Emms and Kelly 2015, 2019) using the 12 Trypanosomatid species were realigned using MAFFT v.7.470 (Katoh and Standley 2013), with the flag ‐‐anysymbol to preserve internal stop codons. Then, the protein coordinates that correspond to each segregating site in the *L. donovani* East Africa population were identified, and the proportion of ortholog proteins that contained the *L. donovani* reference or the alternative allele was estimated. We filtered the SNP data to only maintain positions at which at least one of the amino acid alleles (reference or alternate) was present in at least 80% of the proteins from trypanosomatids, and this amino acid was assumed as the ancestral variant. Next, we classified the potential impact of the nonsynonymous SNPs based on their BLOSUM 62 matrix substitution scores. BLOSUM 62 scores from −3 to −1 were classified as highly impactful amino acid replacements, whereas BLOSUM 62 scores from zero to three were classified as low-impactful amino acid changes.

The DAF corresponds to the population allele frequency of the derived (nonancestral) allele, estimated by counting the number of alleles in the 113 *L. donovani* isolates that correspond to the derived allele and dividing by the total number of alleles in the position; this is a population estimate.

The DARD corresponds to the read depth of the derived allele, estimated individually for each SNP in each of the 113 isolates. It is calculated, for each SNP, as the ratio between the read depth of the alternate allele divided by the total read depth in the position. In this scenario, for the tetrasomic TASC (Chr 31), a DARD of zero means that all four copies of the chromosome have an ancestral allele for that SNP in that isolate, whereas DARDs of 0.25, 0.5, 0.75, and one correspond to one, two, three, and four chromosomal copies having the derived allele. For disomic chromosomes, we mostly expect DARDs of zero, 0.5, and one, meaning, respectively, homozygous ancestral, heterozygous, and homozygous derived. For each isolate, we classified each SNP based on DARD values, in which for each isolate, DARD values > 0.95 were considered as homozygous derived, DARD values between 0.95 and 0.05 as heterozygous, and DARD values < 0.05 as homozygous ancestral. Comparisons of DAF and DARD among and between groups were performed using Mann–Whitney *U* test.

### Evaluation of gene functions in consistently duplicated chromosomes

The evaluation of biological relevant functions in chromosomes that were consistently with extra copies was performed by manual evaluation of gene annotations and by gene set enrichment analysis by ontology (GESEA). This analysis was performed with the representative well-annotated *L. donovani* (BPK282A1), *T. brucei* (TREU927), and *T. cruzi* (CL Brener) genomes, as described in the Supplemental Methods. Three comparisons were made. (1) Shared in all species: genes in orthogroups with members from *L. donovani* Chr 31, from *T. cruzi* Chr 31, and in the interval 1.0–1.5 Mb in *T. brucei* Chromosome 4 and 2.0–2.5 Mb Chromosome 8 compared with all genes in the three species (all TriTryps). In this comparison, only GOs with at least three annotated genes in the test set were evaluated. (2) Shared between two species: genes that were present in these chromosomes only in two species compared with all genes (sets: Leishmania_Tbrucei, Leishmania_Tcruzi, Tbrucei_Tcruzi). In this comparison, only GOs with at least two annotated genes in the test set were evaluated. (3) Exclusive genes: genes from the aforementioned chromosomes that are exclusive to each species (*Leishmania*, *T. brucei*, *T. cruzi*).

### Software availability

The R scripts are available at GitHub (https://github.com/jaumlrc/Ancestral-aneuploidy-in-Trypanosomatids) and as Supplemental Code.

## Supplementary Material

Supplement 1

Supplement 2

Supplement 3

Supplement 4

Supplement 5

Supplement 6

Supplement 7

Supplement 8

Supplement 9

Supplement 10

Supplement 11

Supplement 12

Supplement 13

Supplement 14

Supplement 15

Supplement 16

Supplement 17

Supplement 18

Supplement 19

Supplement 20
