# Supplementary material for: Ancestral aneuploidy and stable chromosomal duplication resulting in differential genome structure and gene expression control in trypanosomatid parasites
Source: Genome Res. 2024 Mar;34(3):441–53. doi: 10.1101/gr.278550.123 (PMC11067883; doi:10.1101/gr.278550.123)
Supplement: Supplement 1 [file Supplemental_Fig_S1.pdf]

Supplemental_Fig_S1.pdf: Sample-by-sample CCNV Heatmaps in all isolates from all clades. Each line corresponds to a different chromosome/scaffold and each column to a different isolate. The chromosome copies are represented by a colour scale from dark-blue (low copy number) to dark-red (high copy number), where euploid chromosomes (~1 copy for each haploid genome copy) are in light blue. The dendrogram on top clusters the samples by UPGMA, based on the Manhattan distance of their chromosome copy numbers.

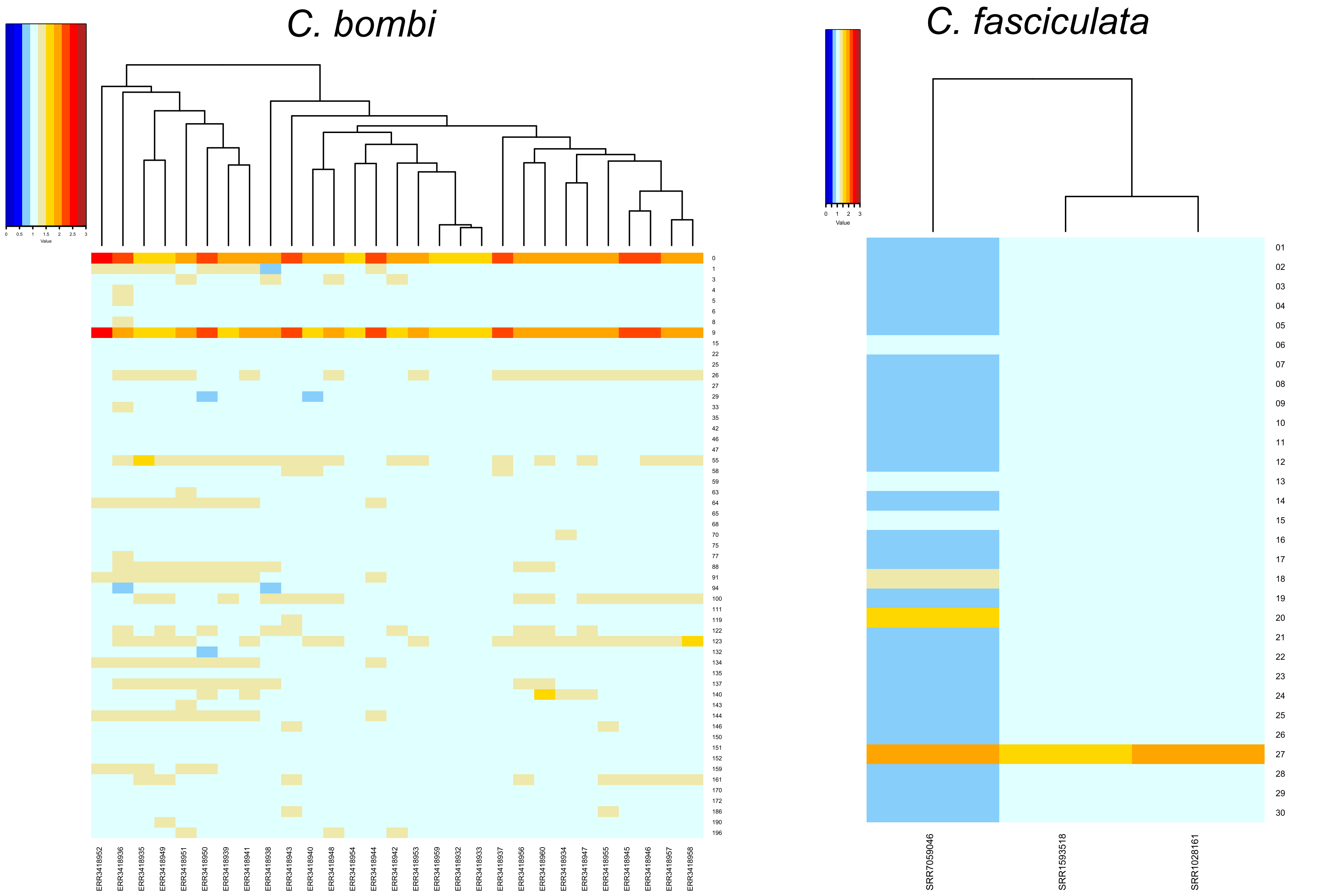

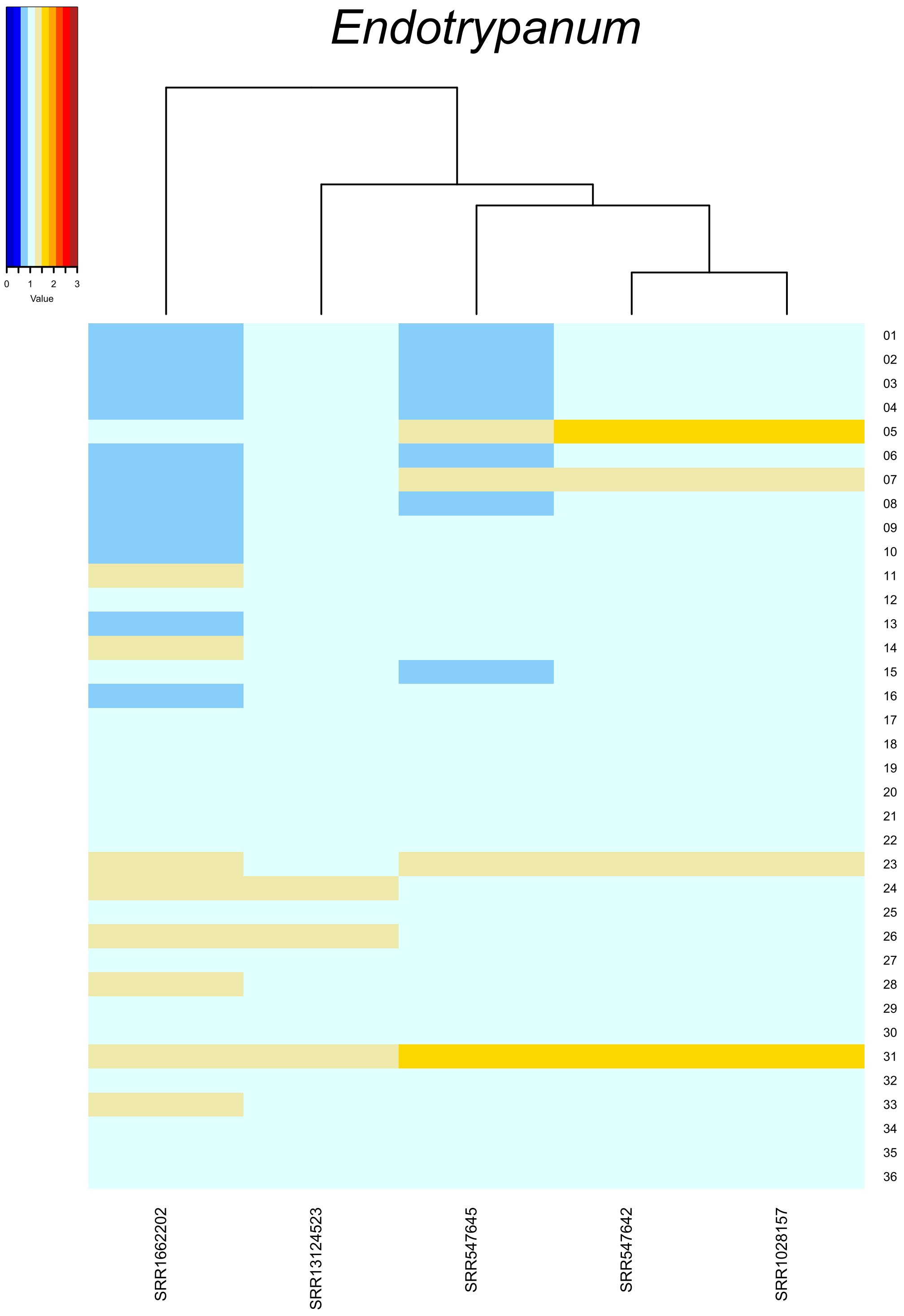

Endotrypanum
0
1
2
3
Value
01
02
03
04
05
06
07
08
09
10
11
12
13
14
15
16
17
18
19
20
21
22
23
24
25
26
27
28
29
30
31
32
33
34
35
36
SRR1662202
SRR13124523
SRR547645
SRR547642
SRR1028157

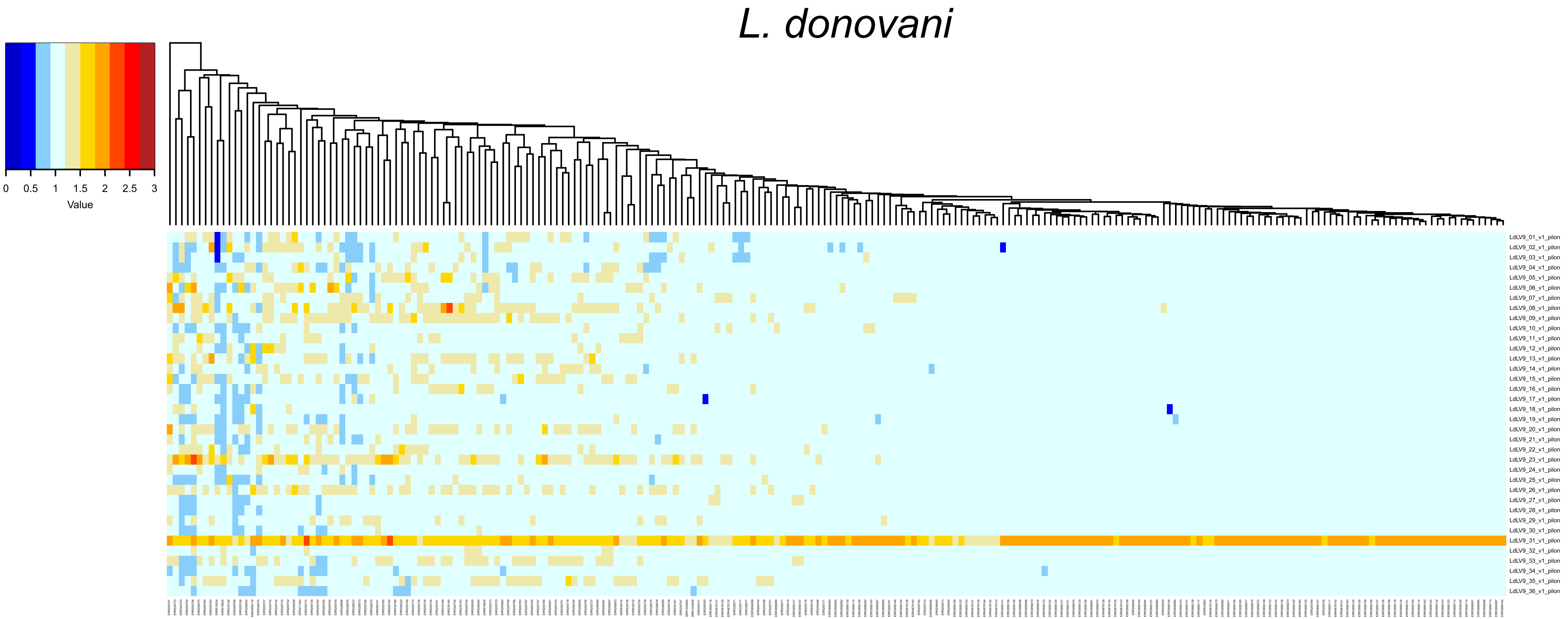
L. donovani
0 0.5 1 1.5 2 2.5 3
Value
LdLV9_01_v1_pilon
LdLV9_02_v1_pilon
LdLV9_03_v1_pilon
LdLV9_04_v1_pilon
LdLV9_05_v1_pilon
LdLV9_06_v1_pilon
LdLV9_07_v1_pilon
LdLV9_08_v1_pilon
LdLV9_09_v1_pilon
LdLV9_10_v1_pilon
LdLV9_11_v1_pilon
LdLV9_12_v1_pilon
LdLV9_13_v1_pilon
LdLV9_14_v1_pilon
LdLV9_15_v1_pilon
LdLV9_16_v1_pilon
LdLV9_17_v1_pilon
LdLV9_18_v1_pilon
LdLV9_19_v1_pilon
LdLV9_20_v1_pilon
LdLV9_21_v1_pilon
LdLV9_22_v1_pilon
LdLV9_23_v1_pilon
LdLV9_24_v1_pilon
LdLV9_25_v1_pilon
LdLV9_26_v1_pilon
LdLV9_27_v1_pilon
LdLV9_28_v1_pilon
LdLV9_29_v1_pilon
LdLV9_30_v1_pilon
LdLV9_31_v1_pilon
LdLV9_32_v1_pilon
LdLV9_33_v1_pilon
LdLV9_34_v1_pilon
LdLV9_35_v1_pilon
LdLV9_36_v1_pilon

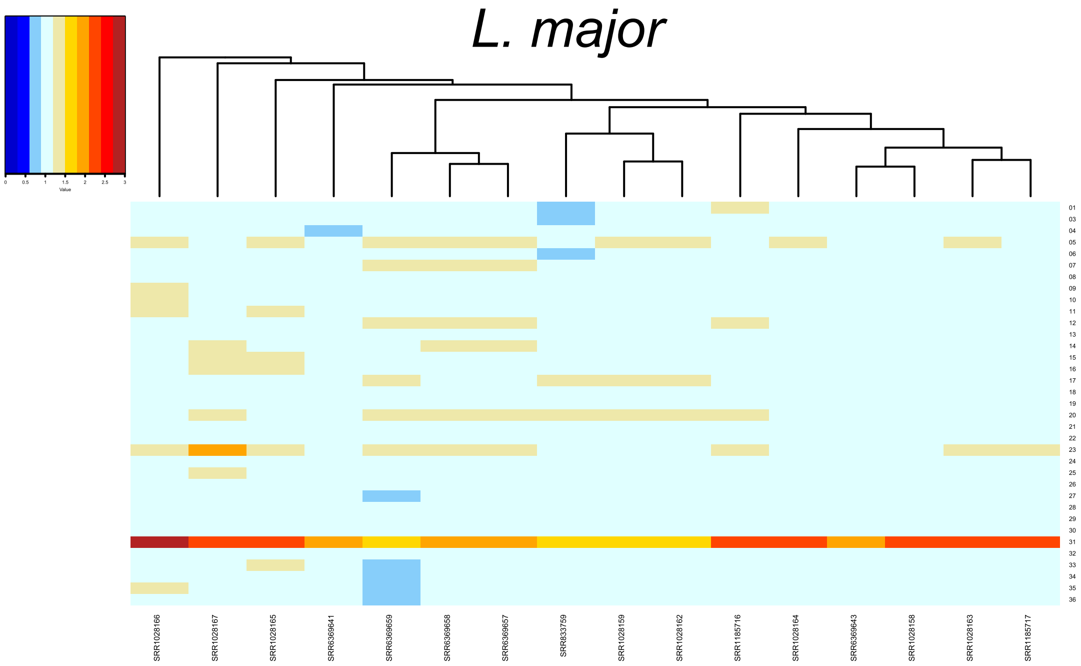
L. major
01
03
04
05
06
07
08
09
10
11
12
13
14
15
16
17
18
19
20
21
22
23
24
25
26
27
28
29
30
31
32
33
34
35
36
SRR1028166
SRR1028167
SRR1028165
SRR6369641
SRR6369659
SRR6369658
SRR6369657
SRR833759
SRR1028159
SRR1028162
SRR1185716
SRR1028164
SRR6369643
SRR1028158
SRR1028163
SRR1185717

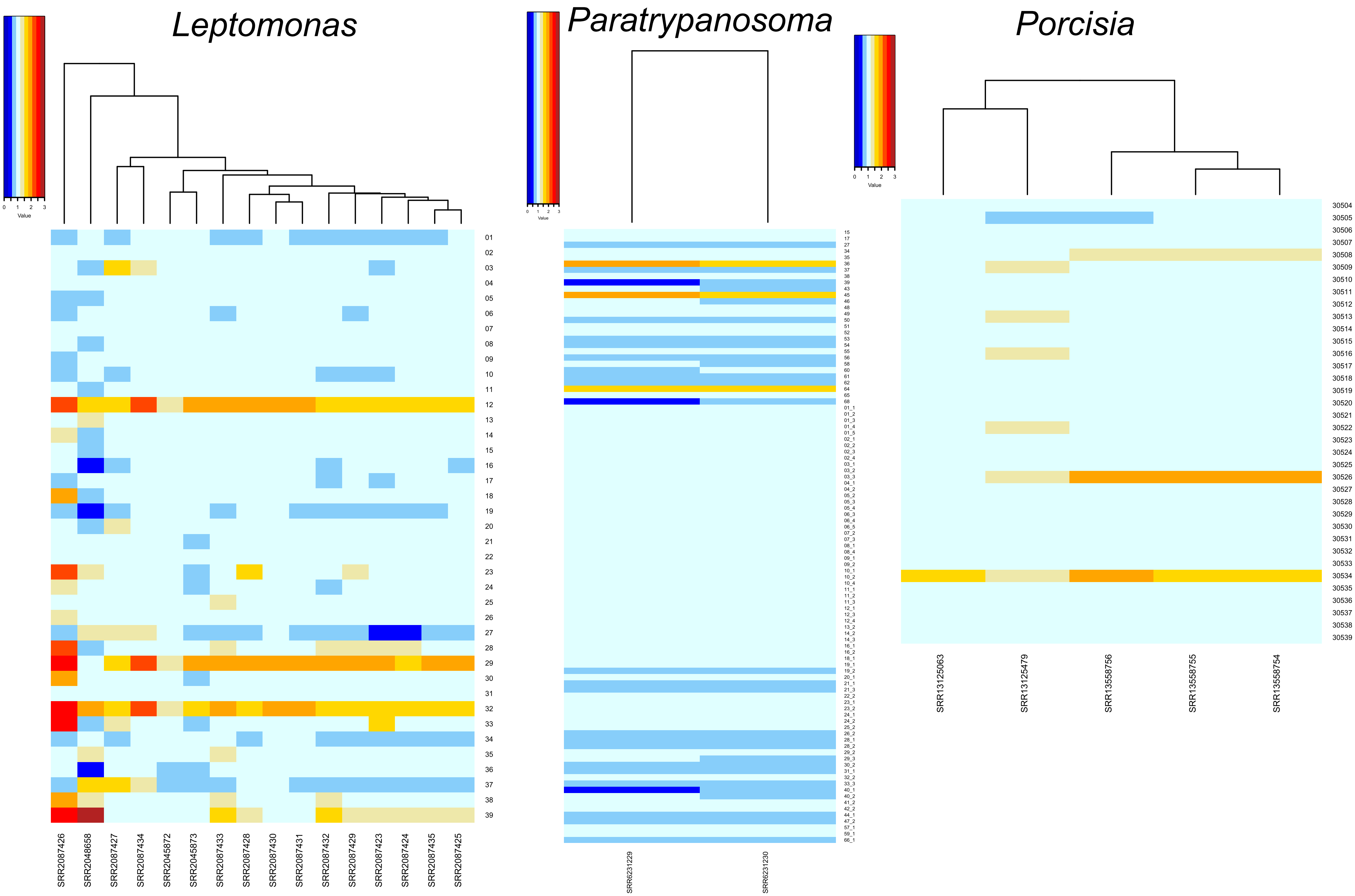
Leptomonas
Paratrypanosoma
Porcisia
Value
0 1 2 3
01
02
03
04
05
06
07
08
09
10
11
12
13
14
15
16
17
18
19
20
21
22
23
24
25
26
27
28
29
30
31
32
33
34
35
36
37
38
39
SRR2087426
SRR2048658
SRR2087427
SRR2087434
SRR2045872
SRR2045873
SRR2087433
SRR2087428
SRR2087430
SRR2087431
SRR2087432
SRR2087429
SRR2087423
SRR2087424
SRR2087435
SRR2087425
SRR6231229
SRR6231230
30504
30505
30506
30507
30508
30509
30510
30511
30512
30513
30514
30515
30516
30517
30518
30519
30520
30521
30522
30523
30524
30525
30526
30527
30528
30529
30530
30531
30532
30533
30534
30535
30536
30537
30538
30539
SRR13125063
SRR13125479
SRR13558756
SRR13558755
SRR13558754

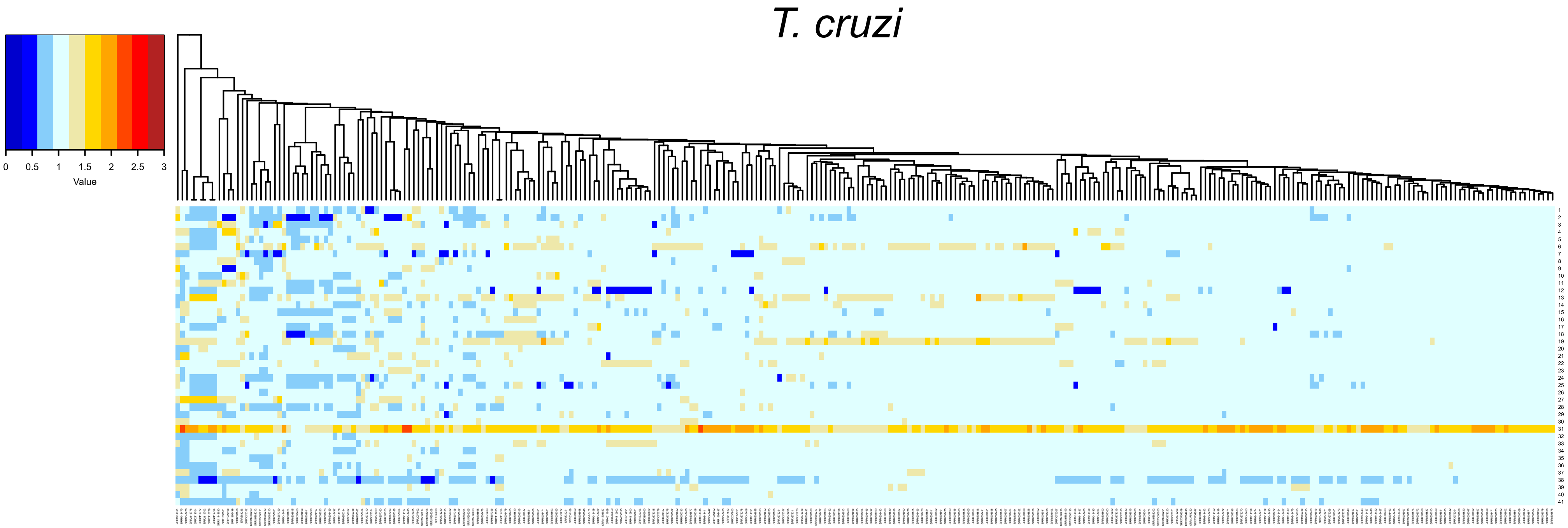
T. cruzi
0 0.5 1 1.5 2 2.5 3
Value
1 2 3 4 5 6 7 8 9 10 11 12 13 14 15 16 17 18 19 20 21 22 23 24 25 26 27 28 29 30 31 32 33 34 35 36 37 38 39 40 41

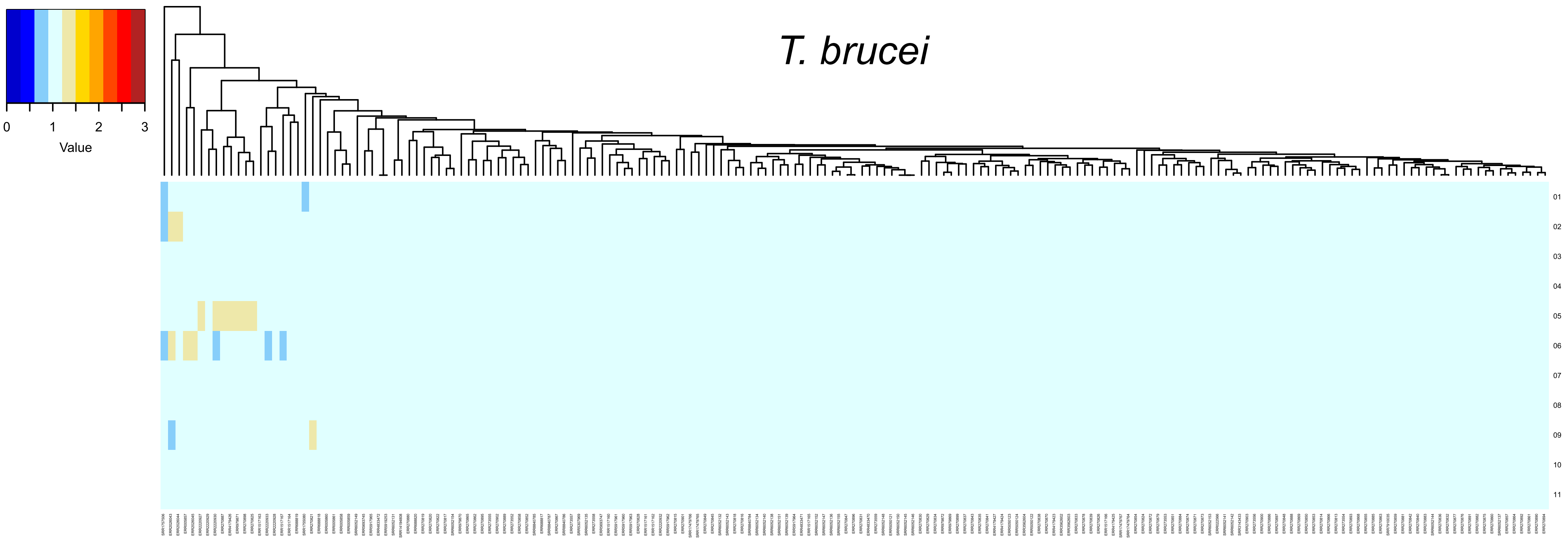
T. brucei
0 1 2 3
Value
01 02 03 04 05 06 07 08 09 10 11

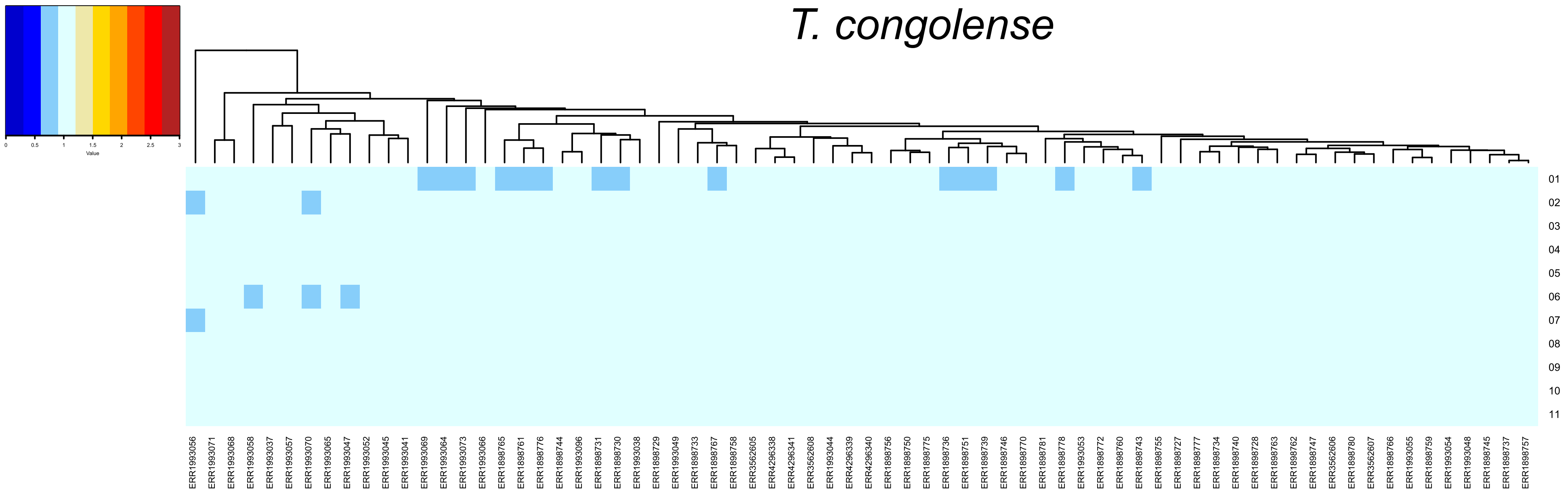
T. congolense
0
0.5
1
1.5
2
2.5
3
Value
01
02
03
04
05
06
07
08
09
10
11
ERR1993056
ERR1993071
ERR1993068
ERR1993058
ERR1993037
ERR1993057
ERR1993070
ERR1993065
ERR1993047
ERR1993052
ERR1993045
ERR1993041
ERR1993069
ERR1993064
ERR1993073
ERR1993066
ERR1898765
ERR1898761
ERR1898776
ERR1898744
ERR1993096
ERR1898731
ERR1898730
ERR1993038
ERR1898729
ERR1993049
ERR1898733
ERR1898767
ERR1898758
ERR3562605
ERR4296338
ERR4296341
ERR3562608
ERR1993044
ERR4296339
ERR4296340
ERR1898756
ERR1898750
ERR1898775
ERR1898736
ERR1898751
ERR1898739
ERR1898746
ERR1898770
ERR1898781
ERR1898778
ERR1993053
ERR1898772
ERR1898760
ERR1898743
ERR1898755
ERR1898727
ERR1898777
ERR1898734
ERR1898740
ERR1898728
ERR1898763
ERR1898762
ERR1898747
ERR3562606
ERR1898780
ERR3562607
ERR1898766
ERR1993055
ERR1898759
ERR1993054
ERR1993048
ERR1898745
ERR1898737
ERR1898757

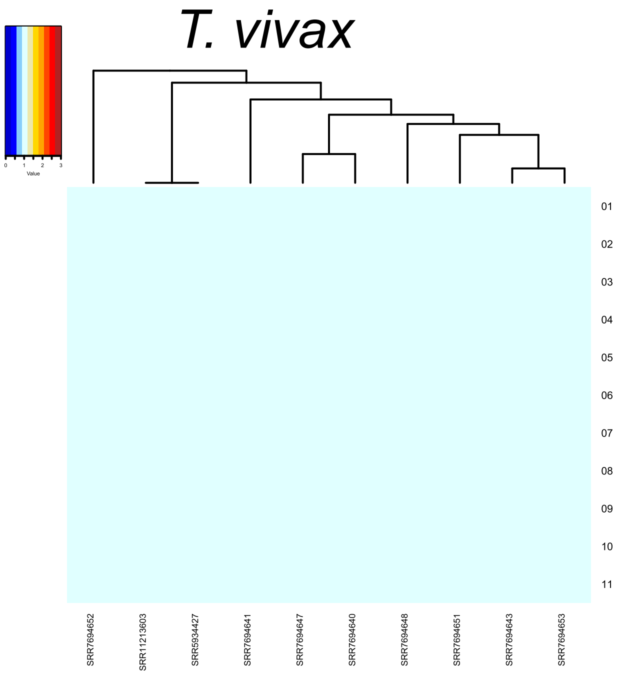
T. vivax
0
1
2
3
Value
01
02
03
04
05
06
07
08
09
10
11
SRR7694652
SRR11213603
SRR5934427
SRR7694641
SRR7694647
SRR7694640
SRR7694648
SRR7694651
SRR7694643
SRR7694653
