# Supplementary material for: Ancestral aneuploidy and stable chromosomal duplication resulting in differential genome structure and gene expression control in trypanosomatid parasites
Source: Genome Res. 2024 Mar;34(3):441–53. doi: 10.1101/gr.278550.123 (PMC11067883; doi:10.1101/gr.278550.123)

Supplemental_Fig_2.pdf: Coefficient of variation (CoV) of gene copies used to estimate chromosomal somy in Trypanosomatids. The CoV of each chromosome, for each isolate, was estimated as the SD of the gene copy numbers in this chromosome divided by the mean gene coverage in the chromosome. Each box corresponds to a different Trypanosomatid clade. TASC chromosomes are highlighted in red. Each dot corresponds to one chromosome from one isolate. The Y axis represents chromosome copy number CoV, and the X axis represents the CCN mean. TASC chromosomes have a low CoV.

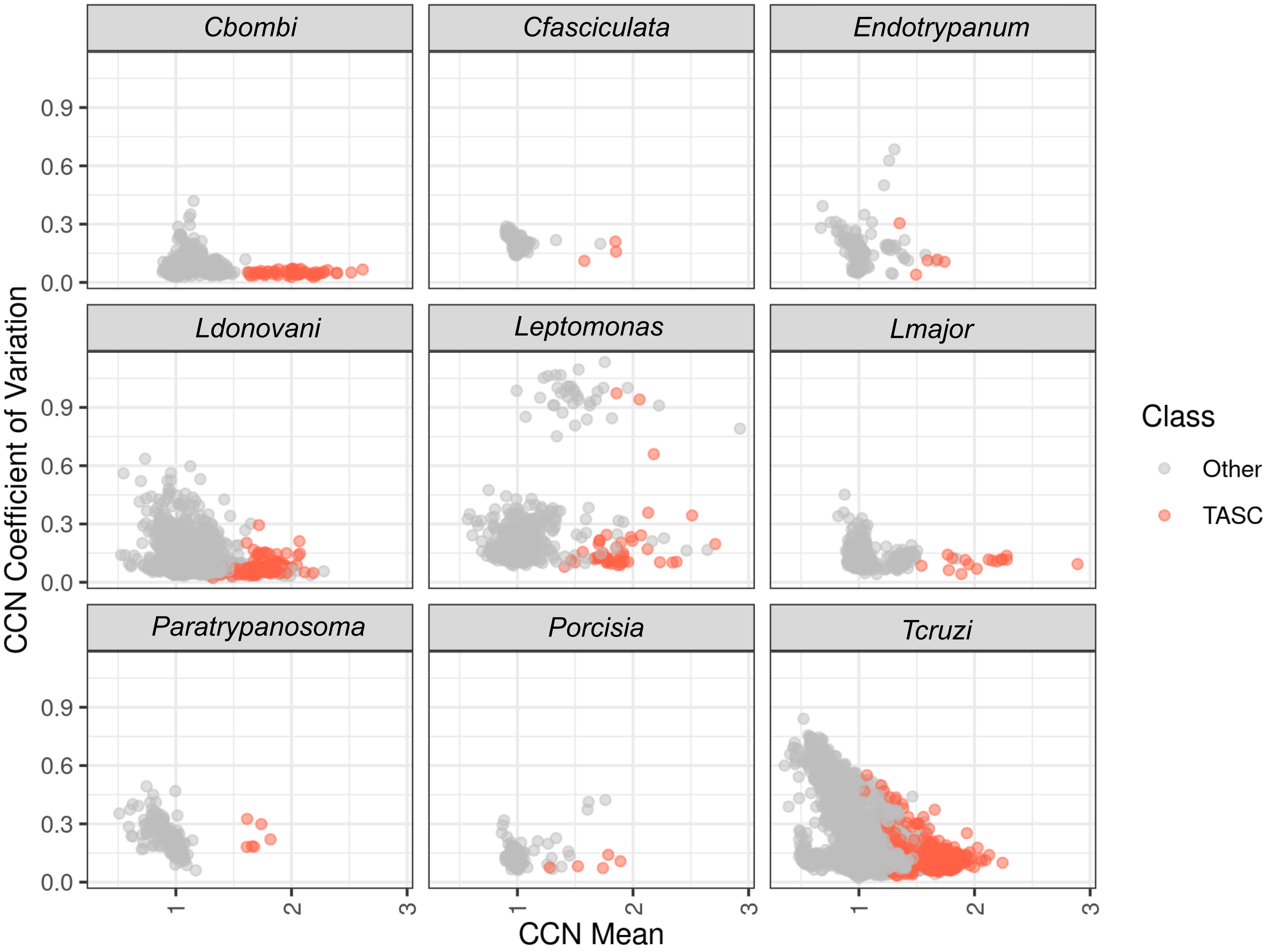

Supplement: Supplement 2 [file Supplemental_Fig_S2.pdf]
