# Supplementary material for: Ancestral aneuploidy and stable chromosomal duplication resulting in differential genome structure and gene expression control in trypanosomatid parasites
Source: Genome Res. 2024 Mar;34(3):441–53. doi: 10.1101/gr.278550.123 (PMC11067883; doi:10.1101/gr.278550.123)
Supplement: Supplement 3 [file Supplemental_Fig_S3.pdf]

Supplemental_Fig_S3.pdf: Proportion of gene sharing between Leish Chr31 and the chromosomes in other species. A) Each panel corresponds to a different species. The Y axis corresponds to the number of shared Orthologs + Paralogs in a given chromosome and the L. major Leish Chr31. Chromosomes with consistent extra copies and syntenic to *L. major* Leish Chr31 are highlighted in gold, while other chromosomes are represented in blue. Chromosomes/scaffolds with less than 10 ortholog genes with Leish Chr31 were “grouped” in the “others” column, in grey. **B)** Proportion of genes in *L. major* Leish Chr31 have ortholog genes in the other species selected chromosomes **C)** The proportion of genes in a given chromosome that have orthologs in *L. major* Leish Chr31 **D)** Table representing the number of genes (total genes in L major chr 31 = 352) in each Leish Chr31 syntenic chromosome and how many of these genes have orthologs in *L. major* Leish Chr31. **Chr_genes**: Number of genes in a given chromosome (or combination of chromosomes, as in *Leptomonas*, *C. bombi* and *P. confusum*). Orthologs: How many of these genes have orthologs/Paralogs in *L. major* Leish Chr31. L. major Orthologs: How many genes in *L. major* Leish Chr31 have orthologs/paralogs with genes in the other species selected chromosomes. **Prop_Lmajor**: The proportion of genes in *L. major* Leish Chr31 have orthologs/paralogs with genes in the other species selected chromosomes, **Prop_Other 31**: The proportion of genes in a given chromosome that have orthologs/Paralogs in *L. major* Leish Chr31.

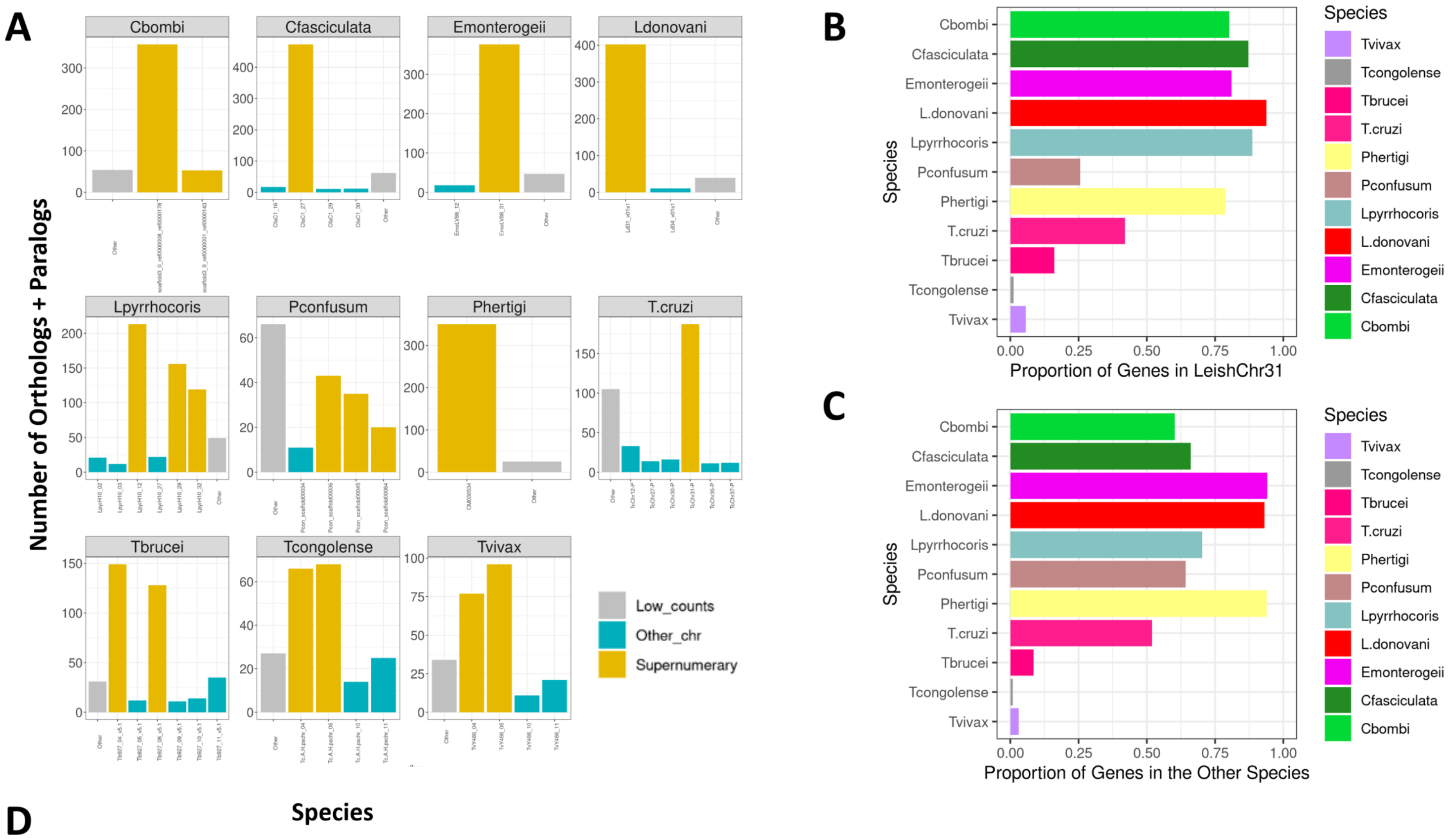

D

Species	Chr	Chr_genes	Ortologs	Lmaj_ortologs	Prop_Lmajor31	Prop_Other31
**L.donovani**	Ld31	332	309	330	0.94	0.93
**Lpyrrhocoris**	LpyrH10_12 - LpyrH10_29 - LpyrH10_32	502	353	312	0.89	0.70
**Cfasciculata**	CfaC1.27	522	345	307	0.87	0.66
**Emonterogeii**	EmoLV88.31	303	285	285	0.81	0.94
**Cbombi**	0 - 9	490	295	282	0.80	0.60
**Phertigi**	CM030534.1	295	277	277	0.79	0.94
**T.cruzi**	TcChr31-P	268	139	148	0.42	0.52
**Pconfusum**	Pc36 - Pc45 - PC64	140	90	90	0.26	0.64
**Tbrucei**	Tb927.04 AND Tb927.08	1440	123	57	0.16	0.09
**Tvivax**	TvY486.04 AND TvY486.08	1247	38	20	0.06	0.03
**Tcongolense**	Tc.A.H.pschr_04 AND Tc.A.H.pschr_08	1421	12	4	0.01	0.01
