# Supplementary material for: Ancestral aneuploidy and stable chromosomal duplication resulting in differential genome structure and gene expression control in trypanosomatid parasites
Source: Genome Res. 2024 Mar;34(3):441–53. doi: 10.1101/gr.278550.123 (PMC11067883; doi:10.1101/gr.278550.123)
Supplement: Supplement 4 [file Supplemental_Fig_S4.pdf]

Supplemental_Fig_S4.pdf: Larger representations of the circa plots from figure 3. Order: *C. bombi; C. fasciculata; Endotrypanum; Leptomonas; Paratrypanosoma; Porcisia; T. cruzi; T. brucei; T. congolense; T. vivax.*

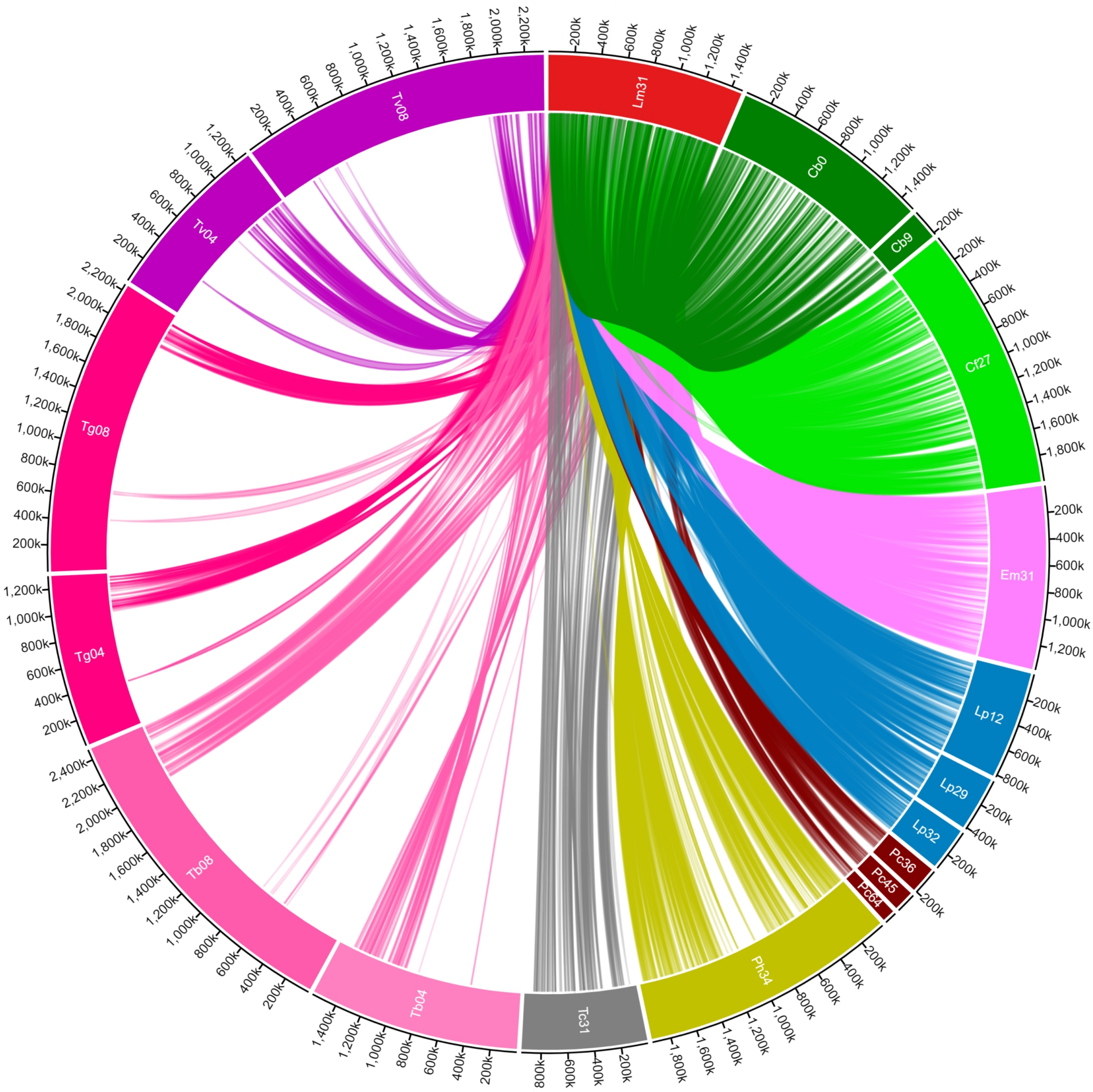

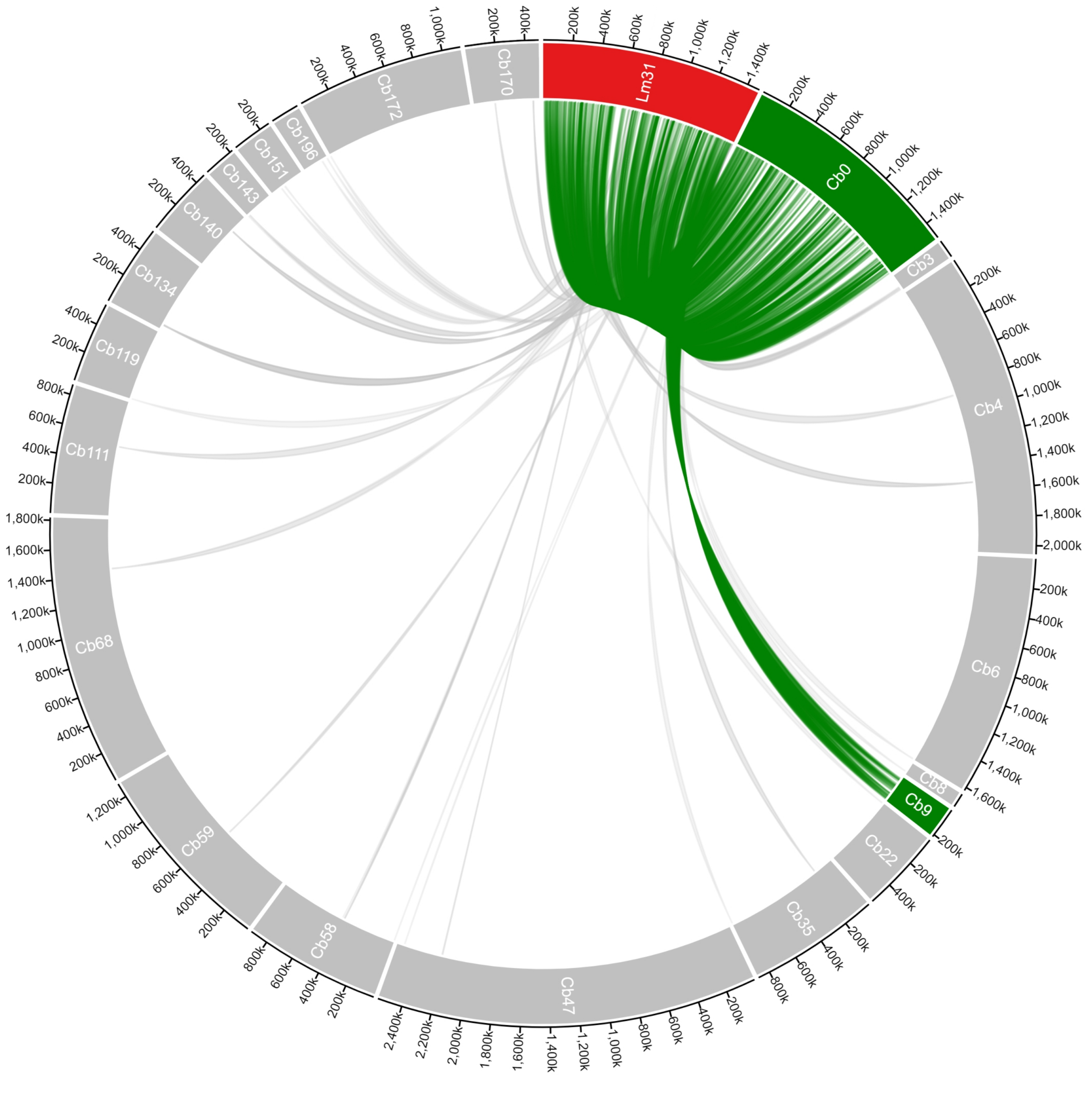
Lm31
Cb0
Cb3
Cb4
Cb6
Cb8
Cb9
Cb22
Cb35
Cb47
Cb58
Cb59
Cb68
Cb111
Cb119
Cb134
Cb140
Cb143
Cb151
Cb196
Cb172
Cb170

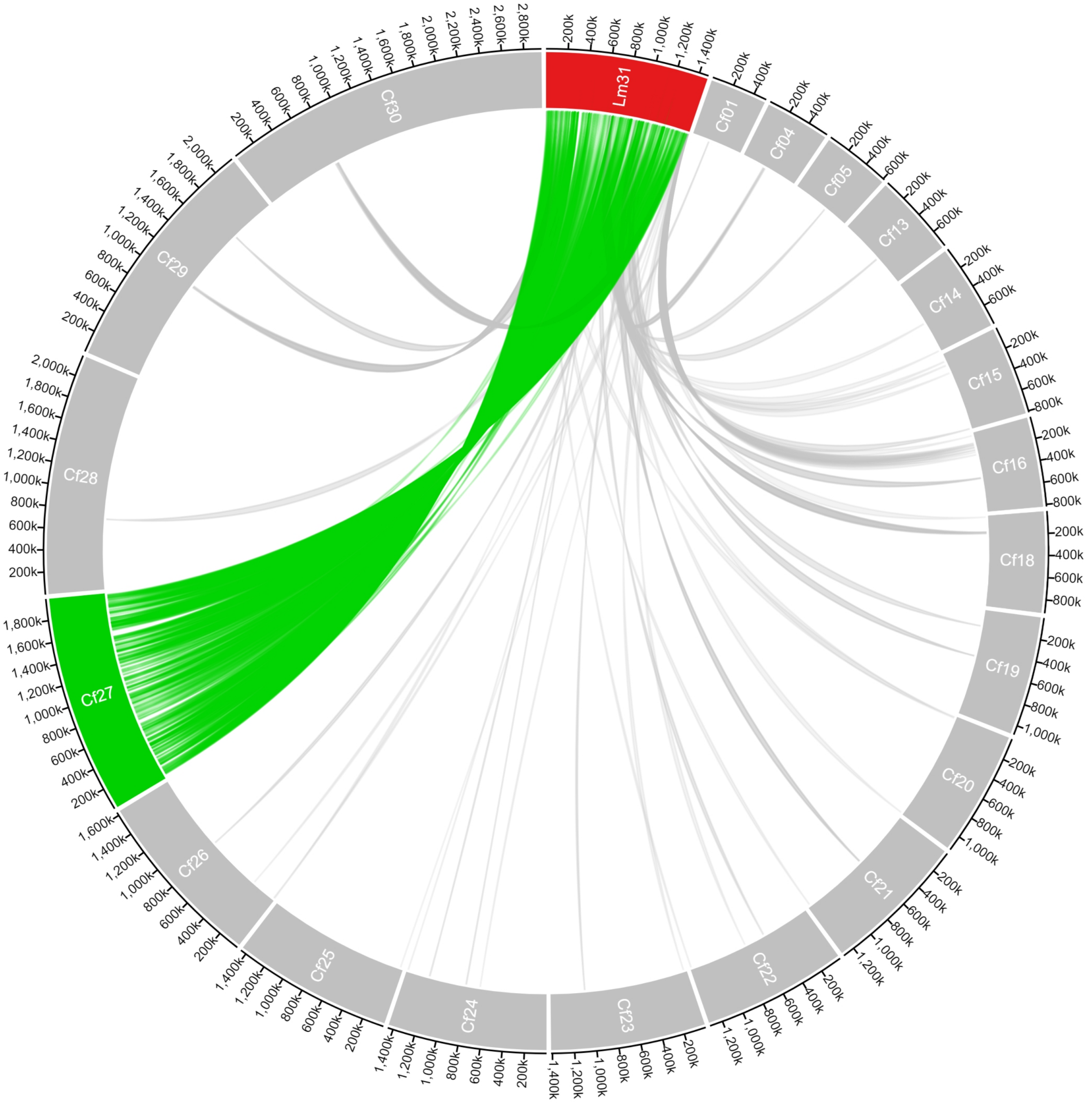
Lm31
Cf01
Cf04
Cf05
Cf13
Cf14
Cf15
Cf16
Cf18
Cf19
Cf20
Cf21
Cf22
Cf23
Cf24
Cf25
Cf26
Cf27
Cf28
Cf29
Cf30

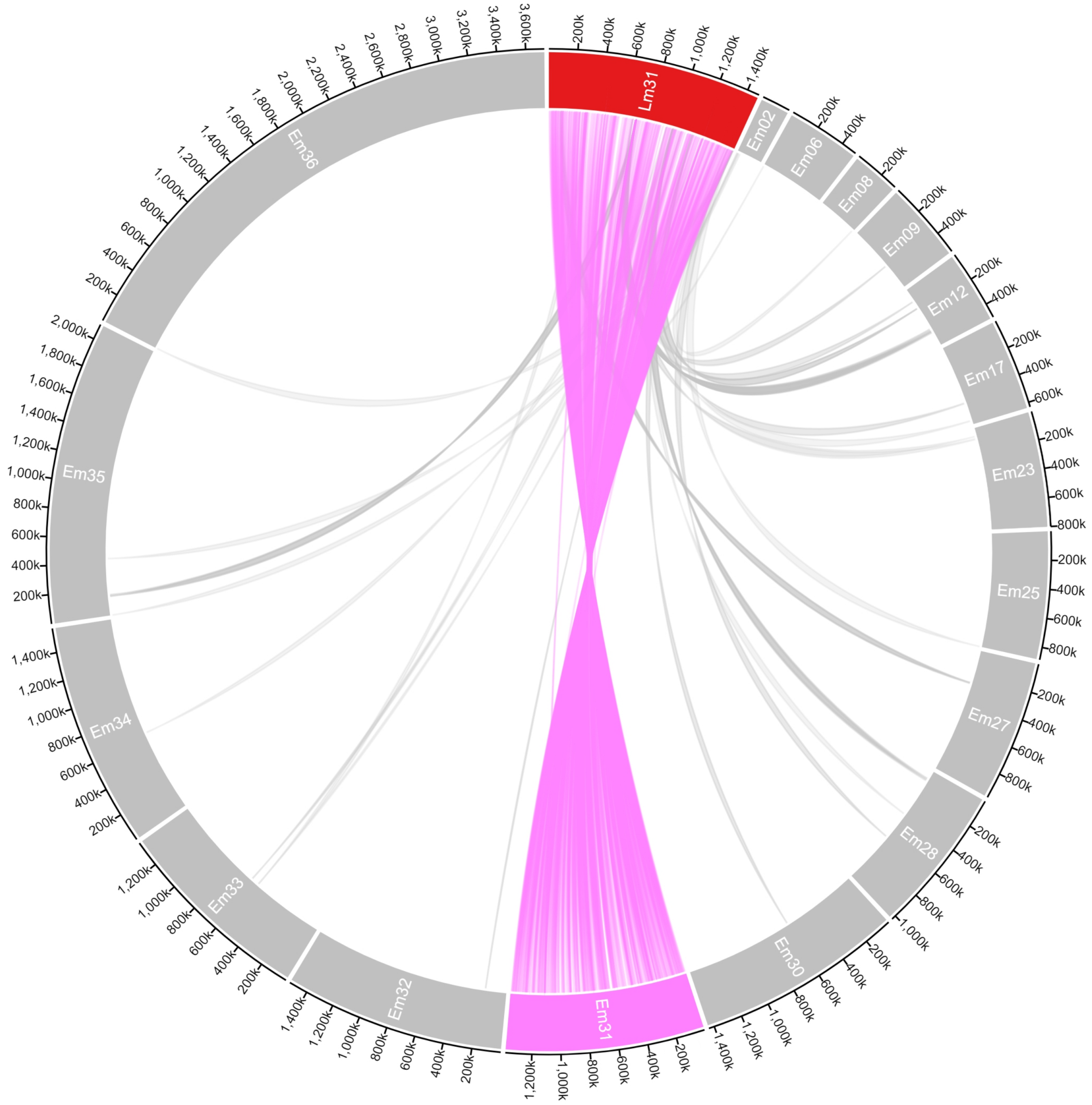
Lm31
Em02
Em06
Em08
Em09
Em12
Em17
Em23
Em25
Em27
Em28
Em30
Em31
Em32
Em33
Em34
Em35
Em36

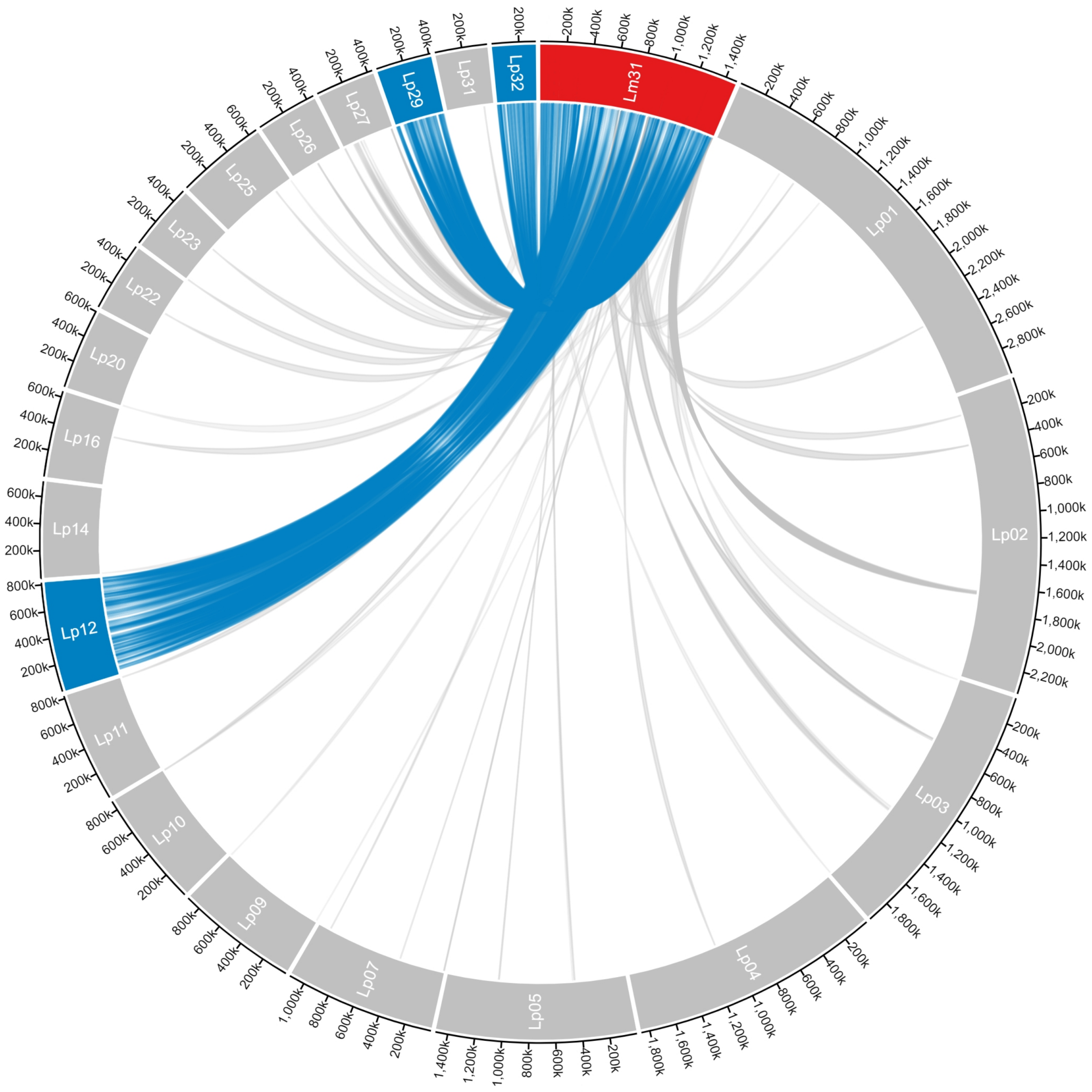

Lm31
Lp01
Lp02
Lp03
Lp04
Lp05
Lp07
Lp09
Lp10
Lp11
Lp12
Lp14
Lp16
Lp20
Lp22
Lp23
Lp25
Lp26
Lp27
Lp29
Lp31
Lp32

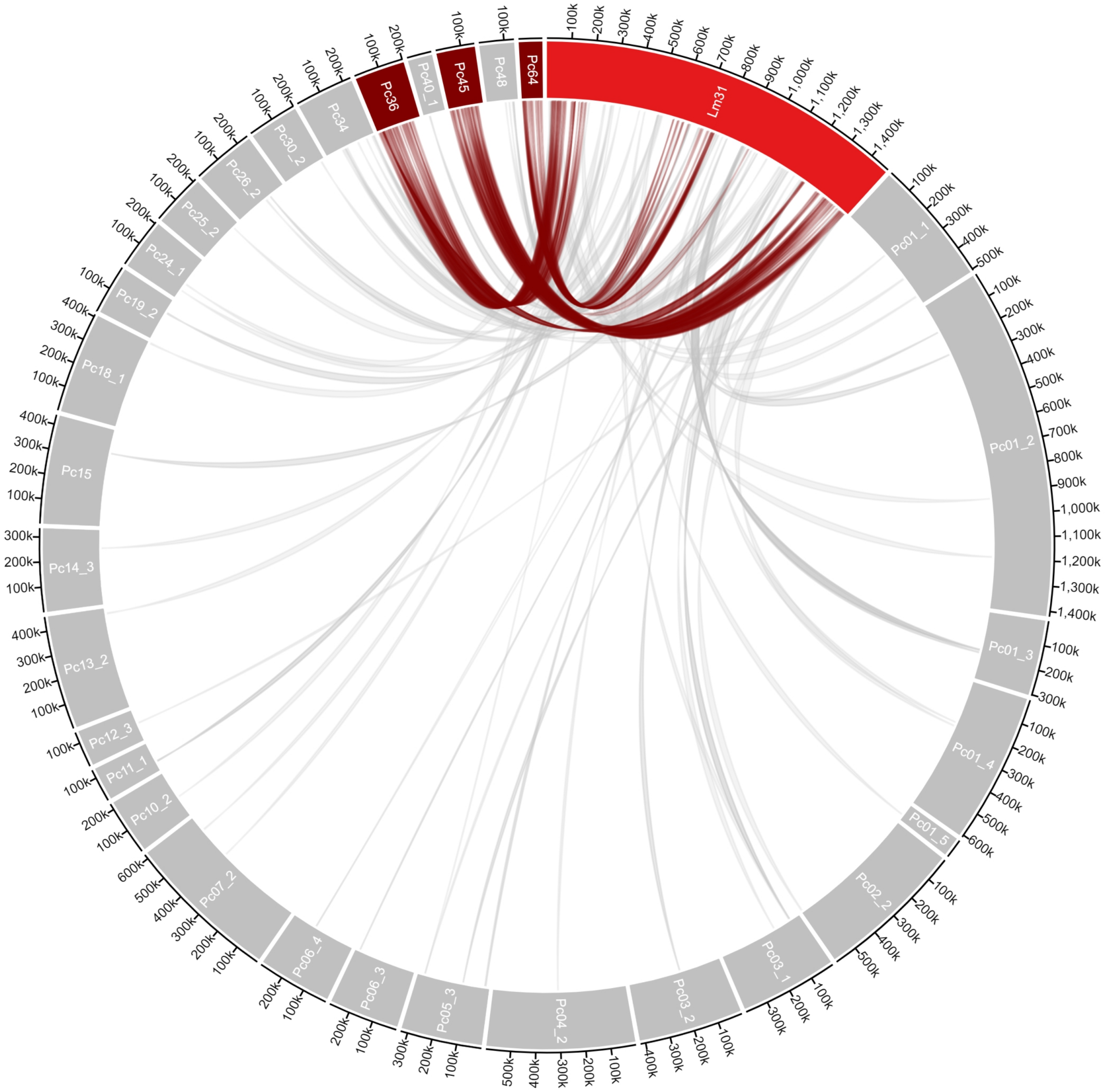

Lm31
Pc01_1
Pc01_2
Pc01_3
Pc01_4
Pc01_5
Pc02_2
Pc03_1
Pc03_2
Pc04_2
Pc05_3
Pc06_3
Pc06_4
Pc07_2
Pc10_2
Pc11_1
Pc12_3
Pc13_2
Pc14_3
Pc15
Pc18_1
Pc19_2
Pc24_1
Pc25_2
Pc26_2
Pc30_2
Pc34
Pc36
Pc40_1
Pc45
Pc48
Pc64

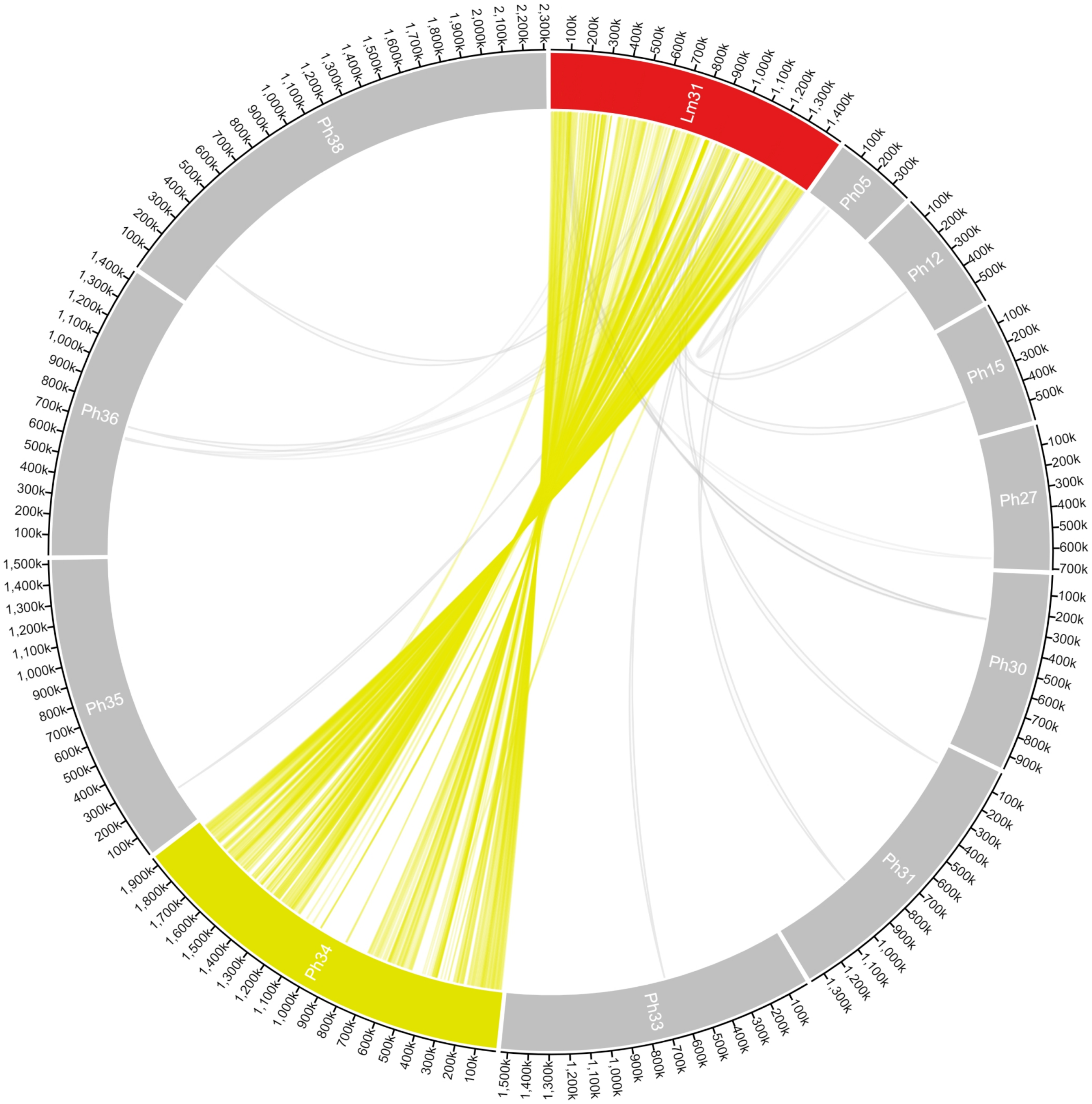
Lm31
Ph05
Ph12
Ph15
Ph27
Ph30
Ph31
Ph33
Ph34
Ph35
Ph36
Ph38

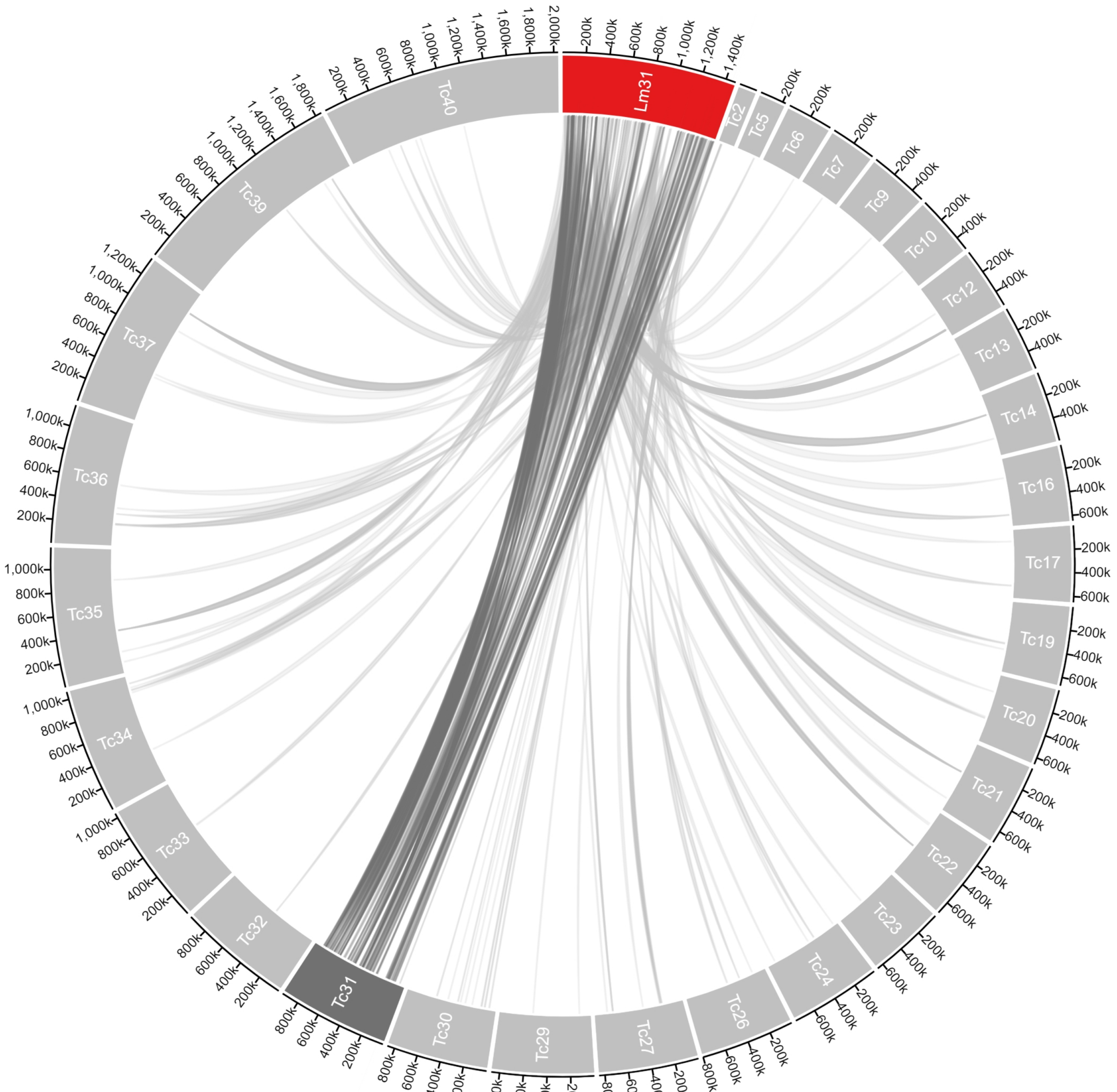

Lm31
Tc2
Tc5
Tc6
Tc7
Tc9
Tc10
Tc12
Tc13
Tc14
Tc16
Tc17
Tc19
Tc20
Tc21
Tc22
Tc23
Tc24
Tc26
Tc27
Tc29
Tc30
Tc31
Tc32
Tc33
Tc34
Tc35
Tc36
Tc37
Tc39
Tc40

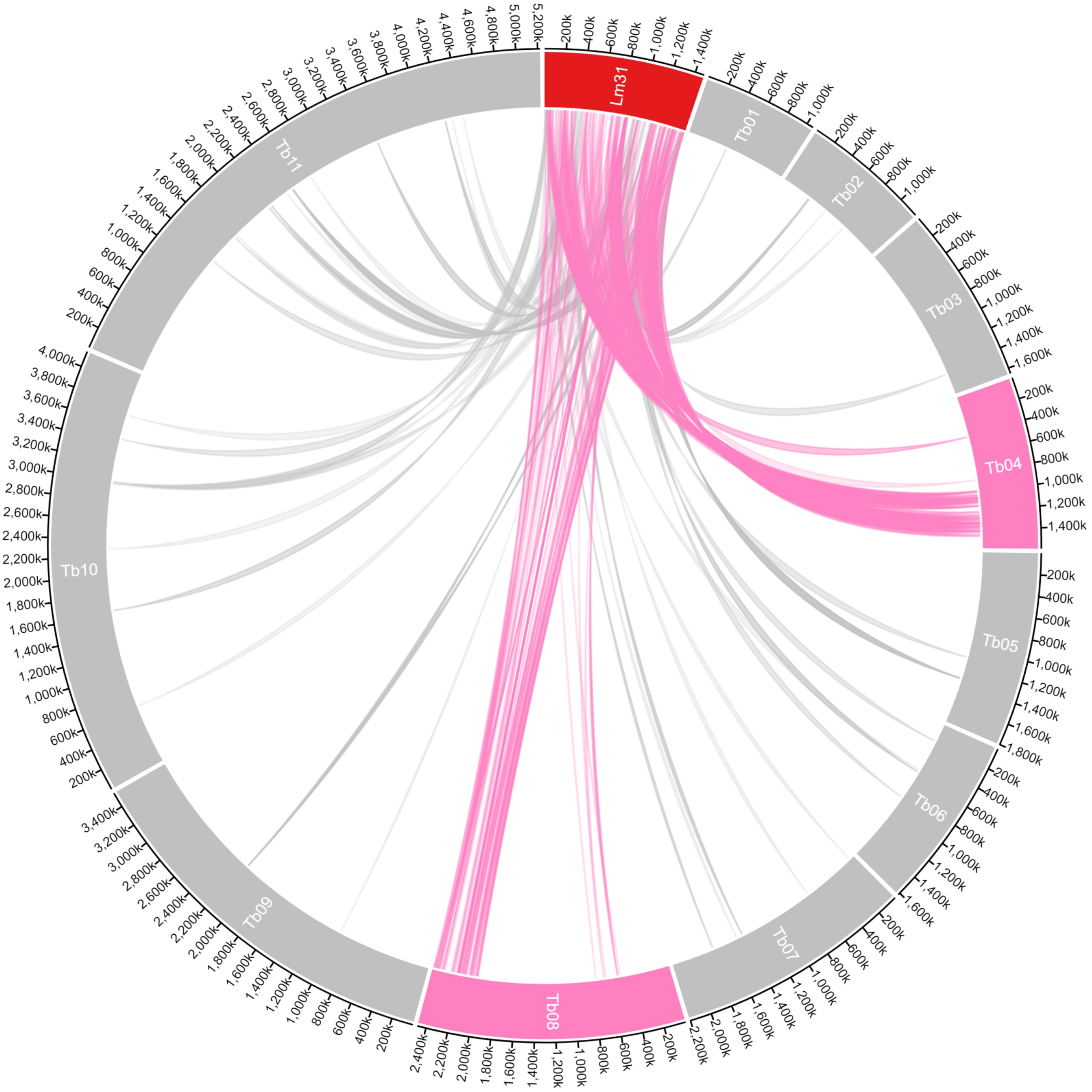
Lm31
Tb01
Tb02
Tb03
Tb04
Tb05
Tb06
Tb07
Tb08
Tb09
Tb10
Tb11

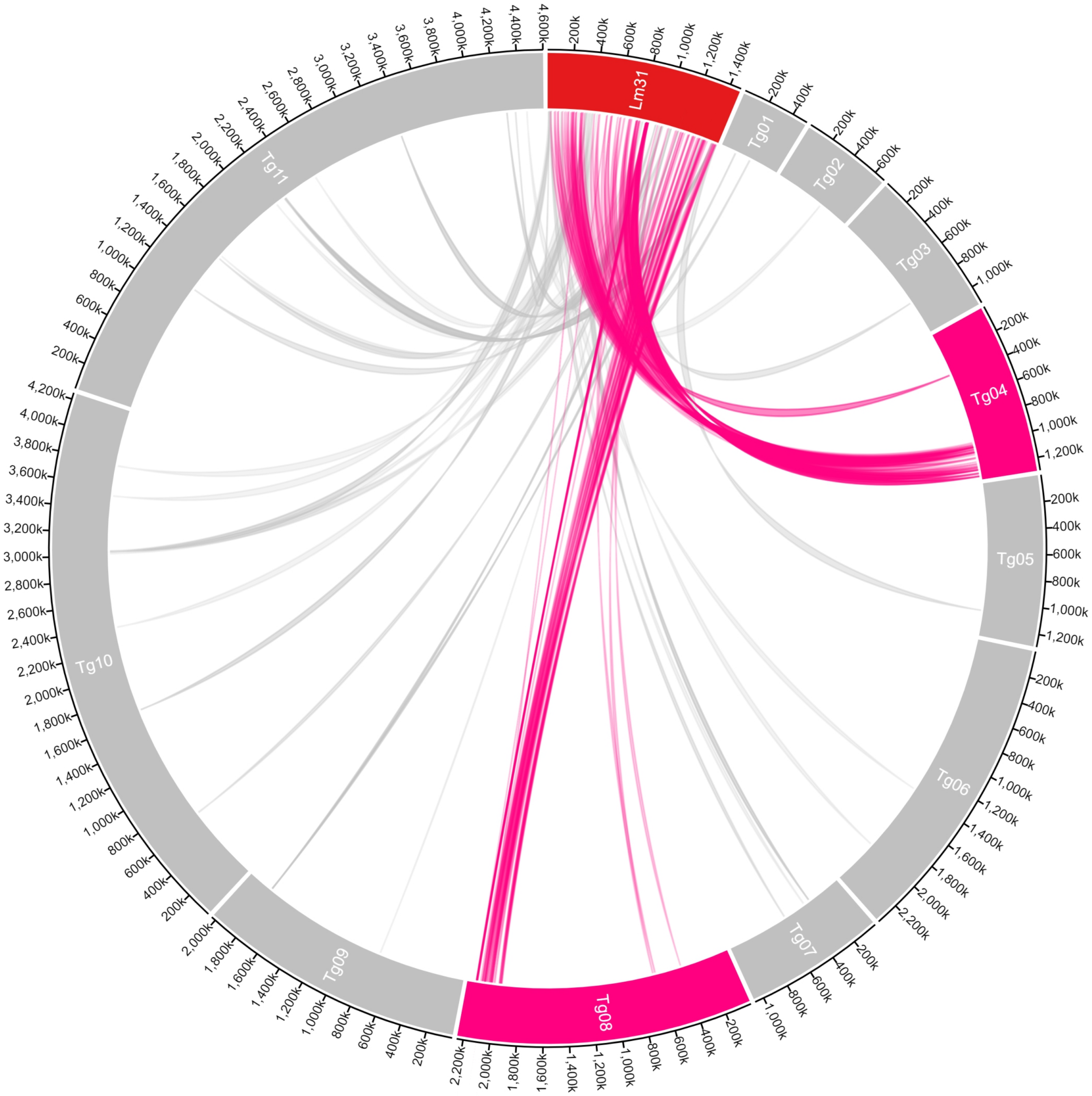
Lm31
Tg01
Tg02
Tg03
Tg04
Tg05
Tg06
Tg07
Tg08
Tg09
Tg10
Tg11

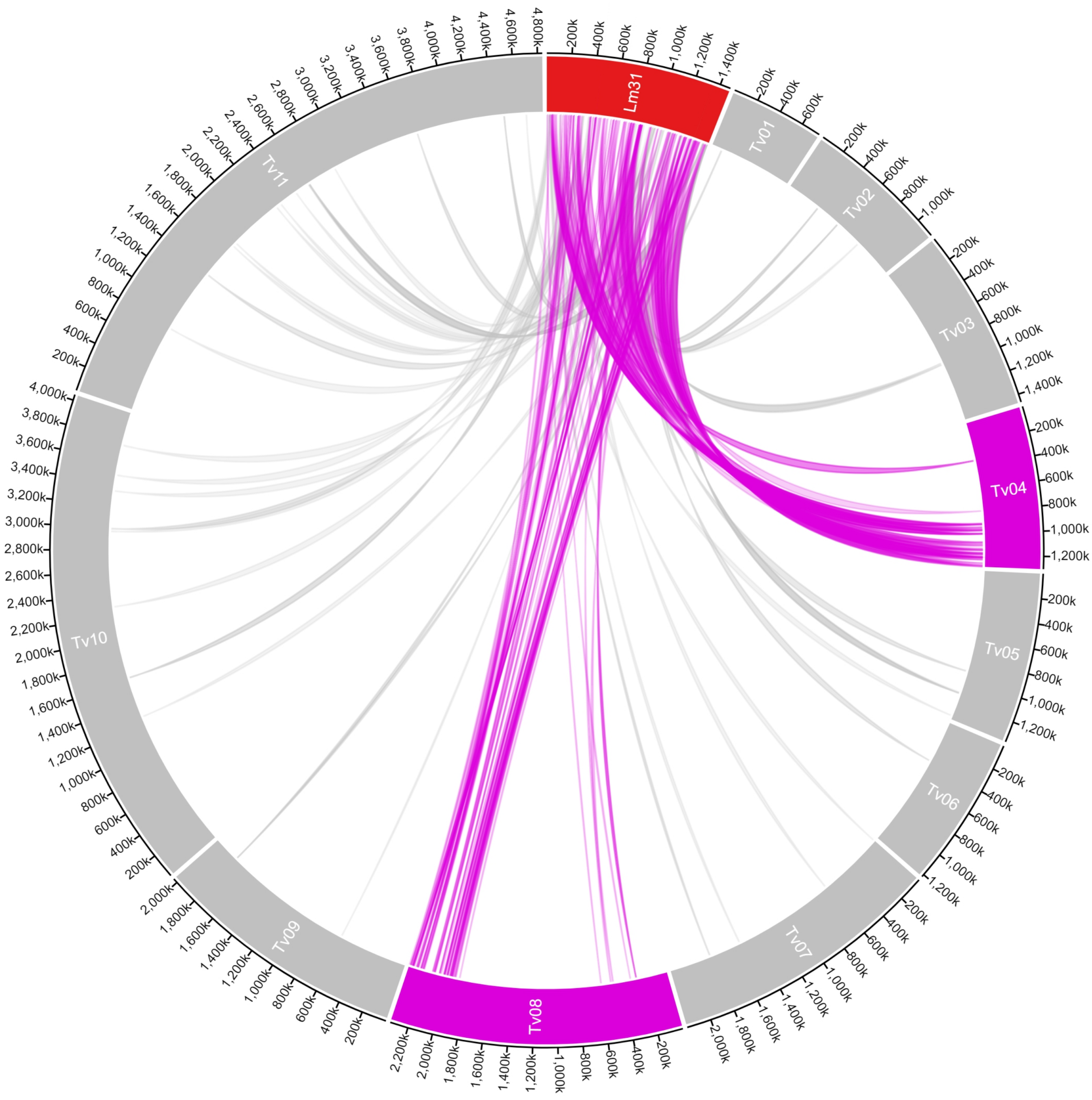
Lm31
Tv01
Tv02
Tv03
Tv04
Tv05
Tv06
Tv07
Tv08
Tv09
Tv10
Tv11
