# Supplementary material for: Ancestral aneuploidy and stable chromosomal duplication resulting in differential genome structure and gene expression control in trypanosomatid parasites
Source: Genome Res. 2024 Mar;34(3):441–53. doi: 10.1101/gr.278550.123 (PMC11067883; doi:10.1101/gr.278550.123)
Supplement: Supplement 5 [file Supplemental_Fig_S5.pdf]

Supplemental_Fig_S5.pdf: Gene disposition and orientation in Trypanosomatids chrs. For each cade, chromosomes are drawn in scale. Genes are represented by black boxes and their orientation by the presence above (plus strand) or below (minus strand) of the chromosomal line.

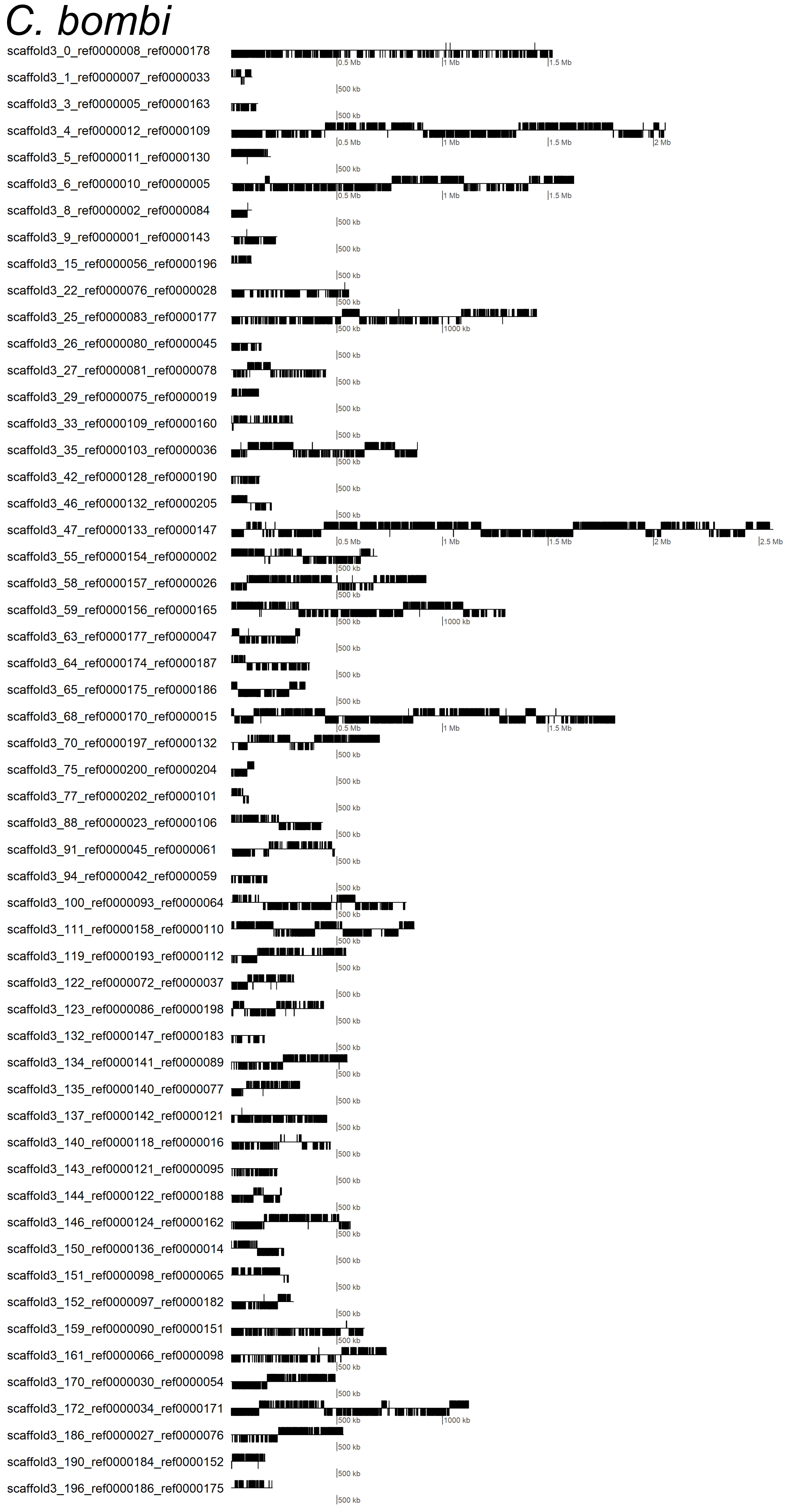

C. fasciculata

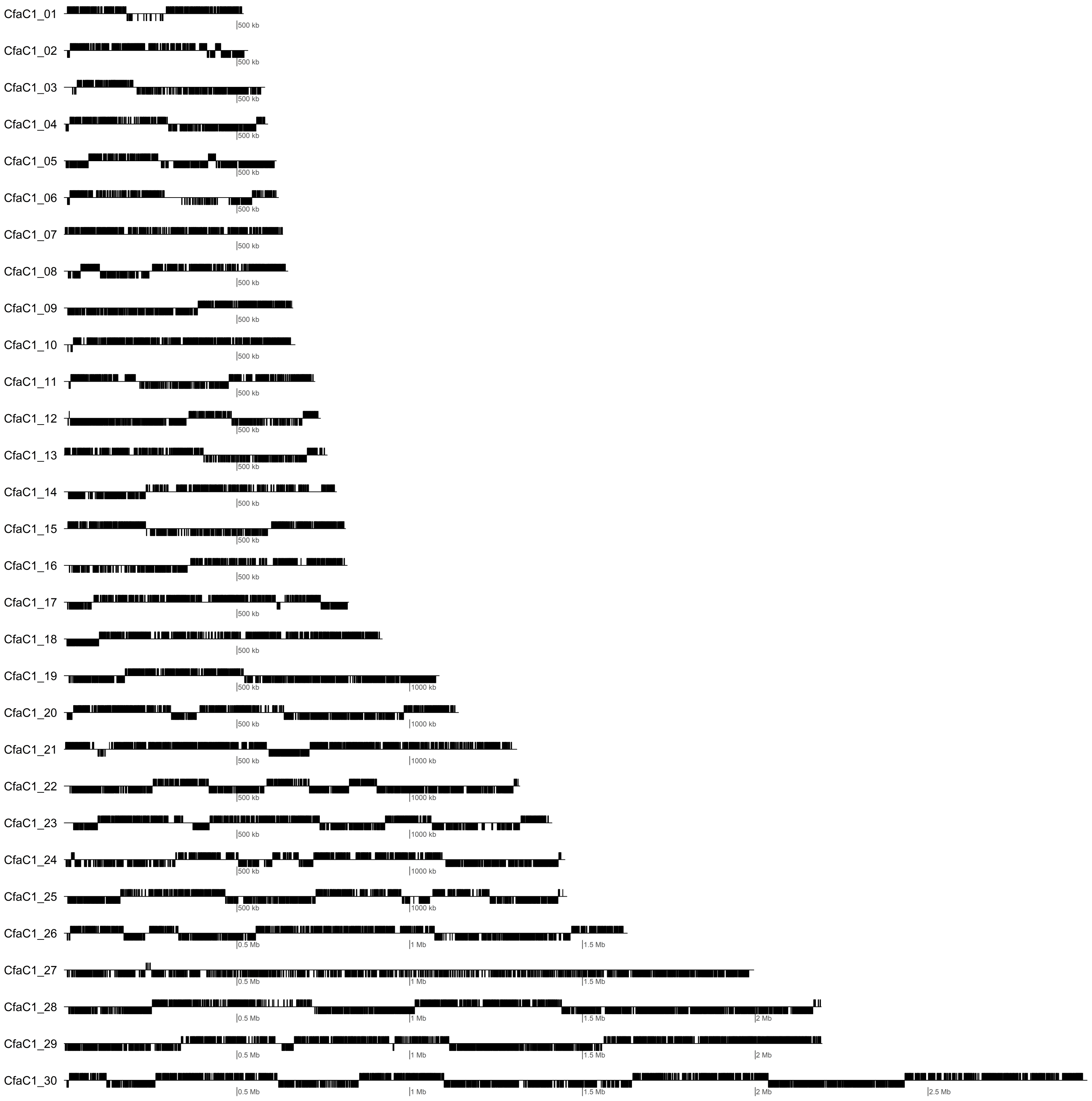

Endotrypanum

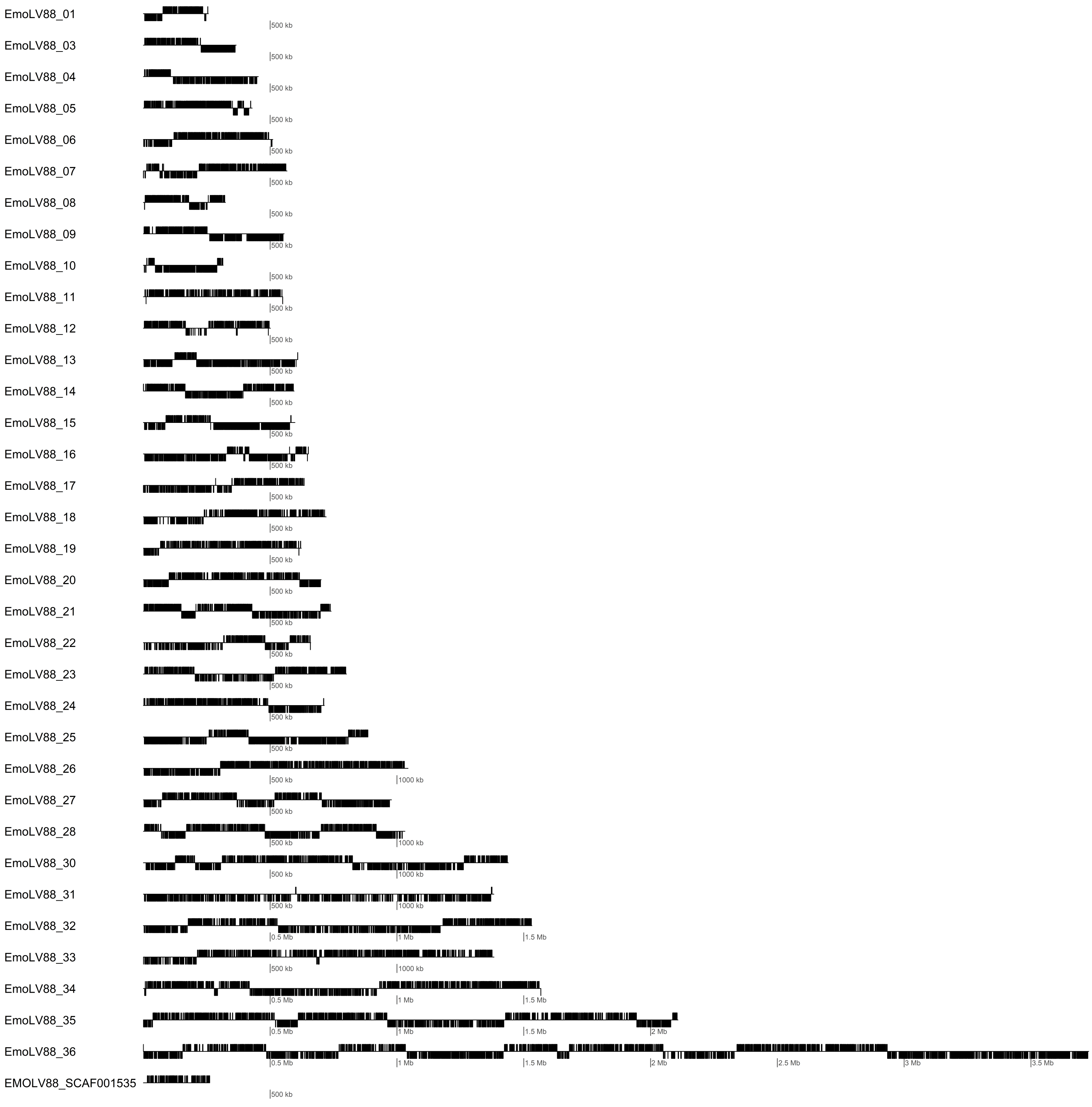

L. donovani

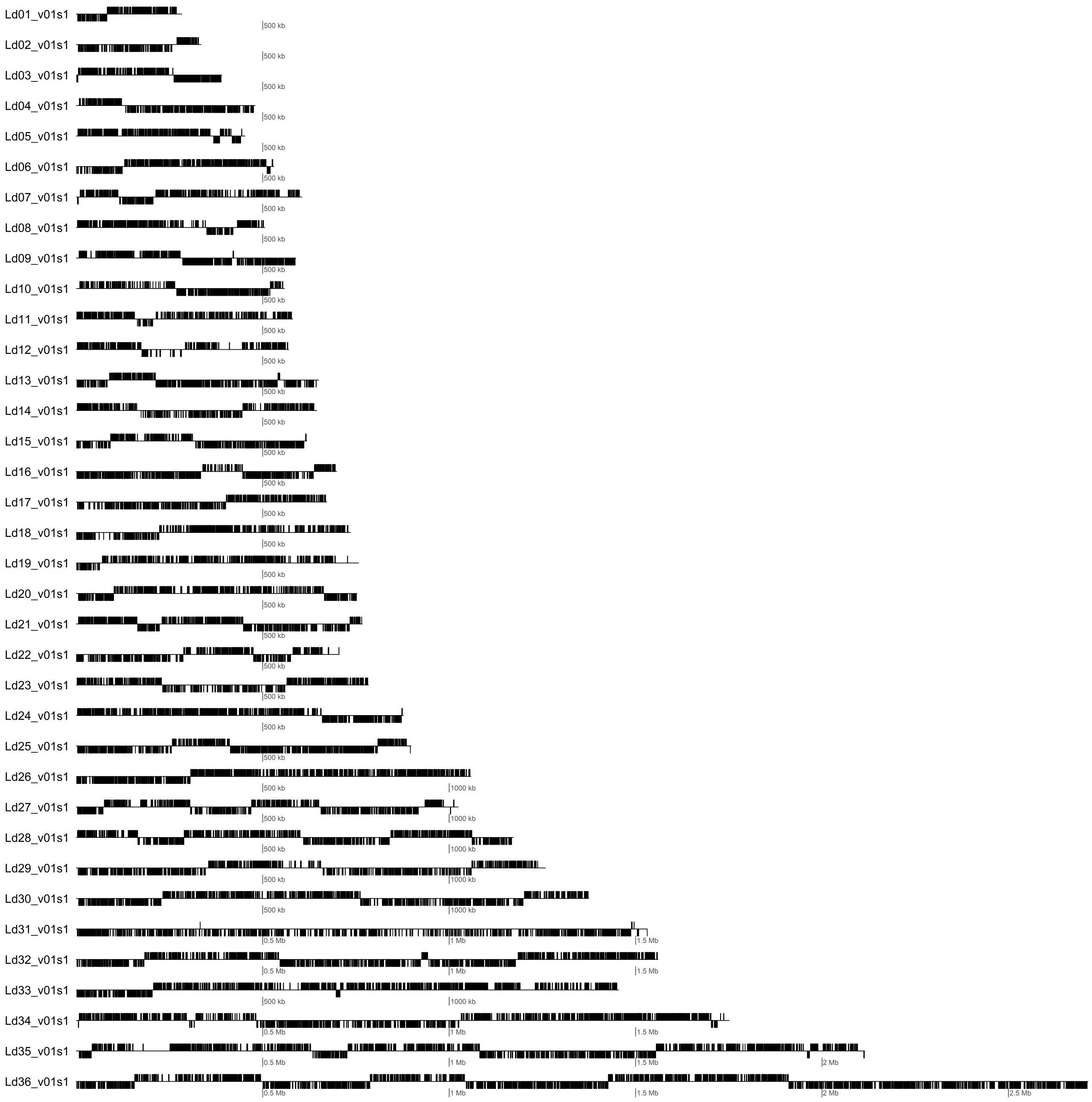

Ld01_v01s1
Ld02_v01s1
Ld03_v01s1
Ld04_v01s1
Ld05_v01s1
Ld06_v01s1
Ld07_v01s1
Ld08_v01s1
Ld09_v01s1
Ld10_v01s1
Ld11_v01s1
Ld12_v01s1
Ld13_v01s1
Ld14_v01s1
Ld15_v01s1
Ld16_v01s1
Ld17_v01s1
Ld18_v01s1
Ld19_v01s1
Ld20_v01s1
Ld21_v01s1
Ld22_v01s1
Ld23_v01s1
Ld24_v01s1
Ld25_v01s1
Ld26_v01s1
Ld27_v01s1
Ld28_v01s1
Ld29_v01s1
Ld30_v01s1
Ld31_v01s1
Ld32_v01s1
Ld33_v01s1
Ld34_v01s1
Ld35_v01s1
Ld36_v01s1
500 kb
1000 kb
0.5 Mb
1 Mb
1.5 Mb
2 Mb
2.5 Mb

L. major

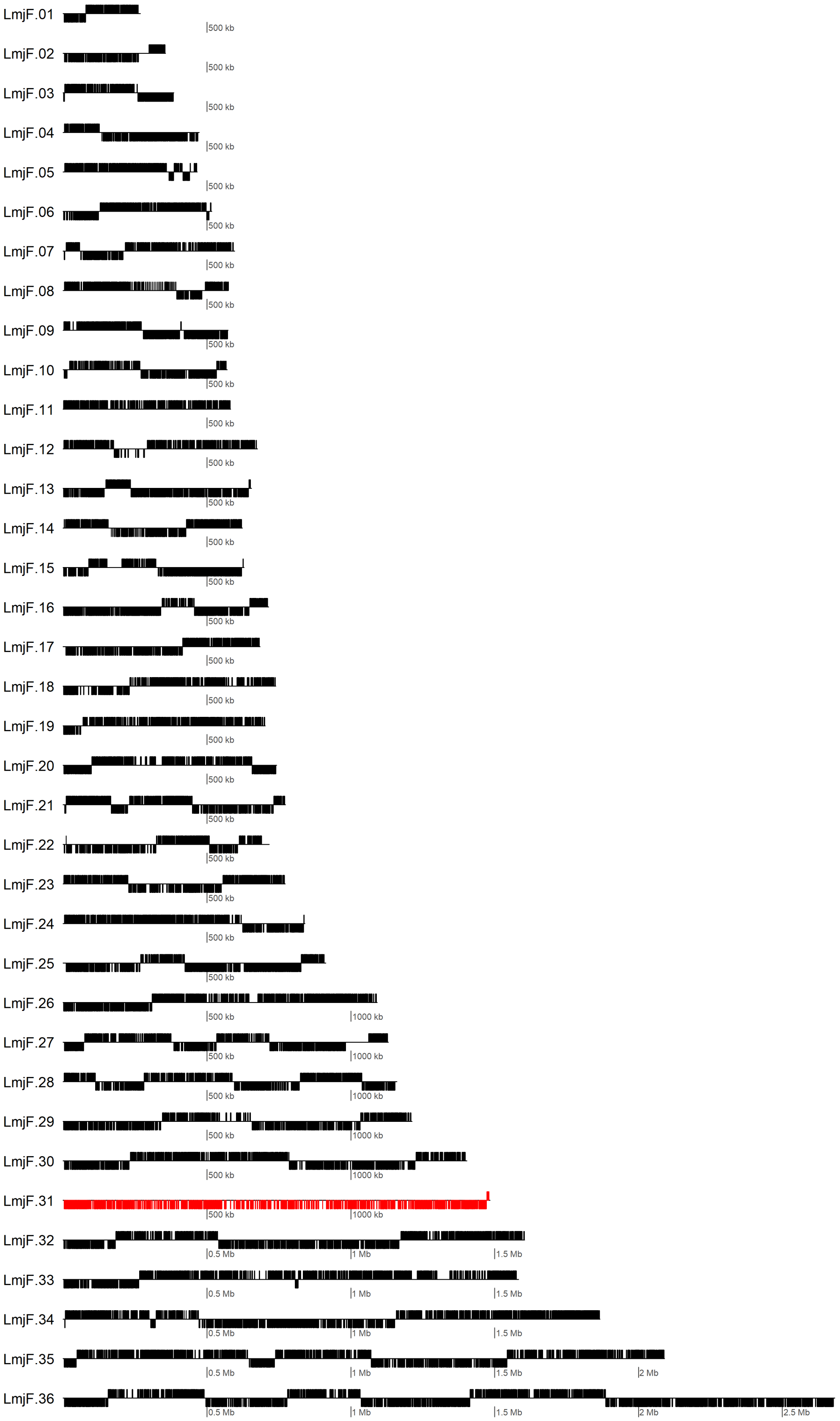

LmjF.01
LmjF.02
LmjF.03
LmjF.04
LmjF.05
LmjF.06
LmjF.07
LmjF.08
LmjF.09
LmjF.10
LmjF.11
LmjF.12
LmjF.13
LmjF.14
LmjF.15
LmjF.16
LmjF.17
LmjF.18
LmjF.19
LmjF.20
LmjF.21
LmjF.22
LmjF.23
LmjF.24
LmjF.25
LmjF.26
LmjF.27
LmjF.28
LmjF.29
LmjF.30
LmjF.31
LmjF.32
LmjF.33
LmjF.34
LmjF.35
LmjF.36
500 kb
1000 kb
0.5 Mb
1 Mb
1.5 Mb
2 Mb
2.5 Mb

Leptomonas

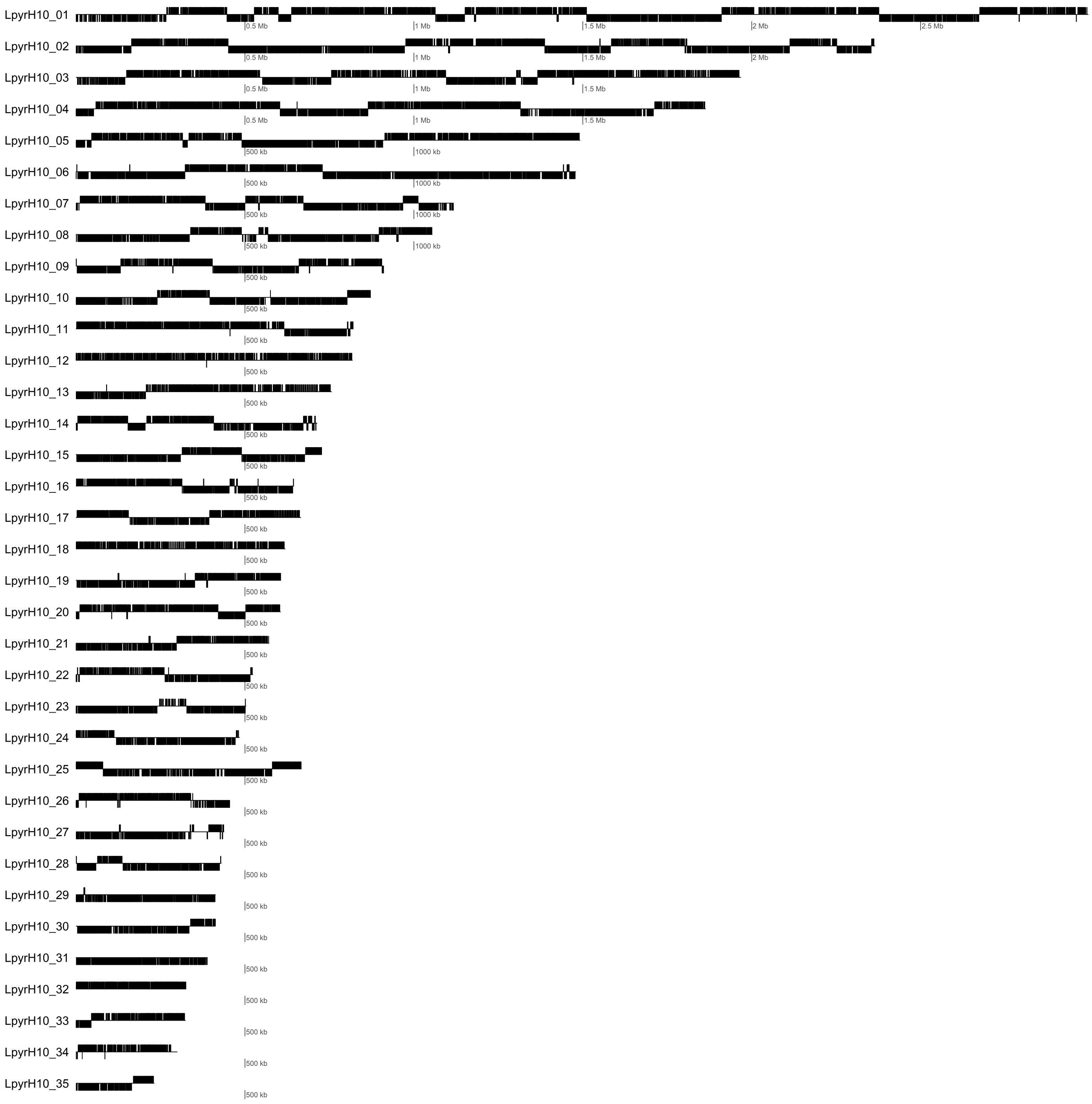
LpyrH10_01
LpyrH10_02
LpyrH10_03
LpyrH10_04
LpyrH10_05
LpyrH10_06
LpyrH10_07
LpyrH10_08
LpyrH10_09
LpyrH10_10
LpyrH10_11
LpyrH10_12
LpyrH10_13
LpyrH10_14
LpyrH10_15
LpyrH10_16
LpyrH10_17
LpyrH10_18
LpyrH10_19
LpyrH10_20
LpyrH10_21
LpyrH10_22
LpyrH10_23
LpyrH10_24
LpyrH10_25
LpyrH10_26
LpyrH10_27
LpyrH10_28
LpyrH10_29
LpyrH10_30
LpyrH10_31
LpyrH10_32
LpyrH10_33
LpyrH10_34
LpyrH10_35
0.5 Mb
1 Mb
1.5 Mb
2 Mb
2.5 Mb
500 kb
1000 kb

P. confusum

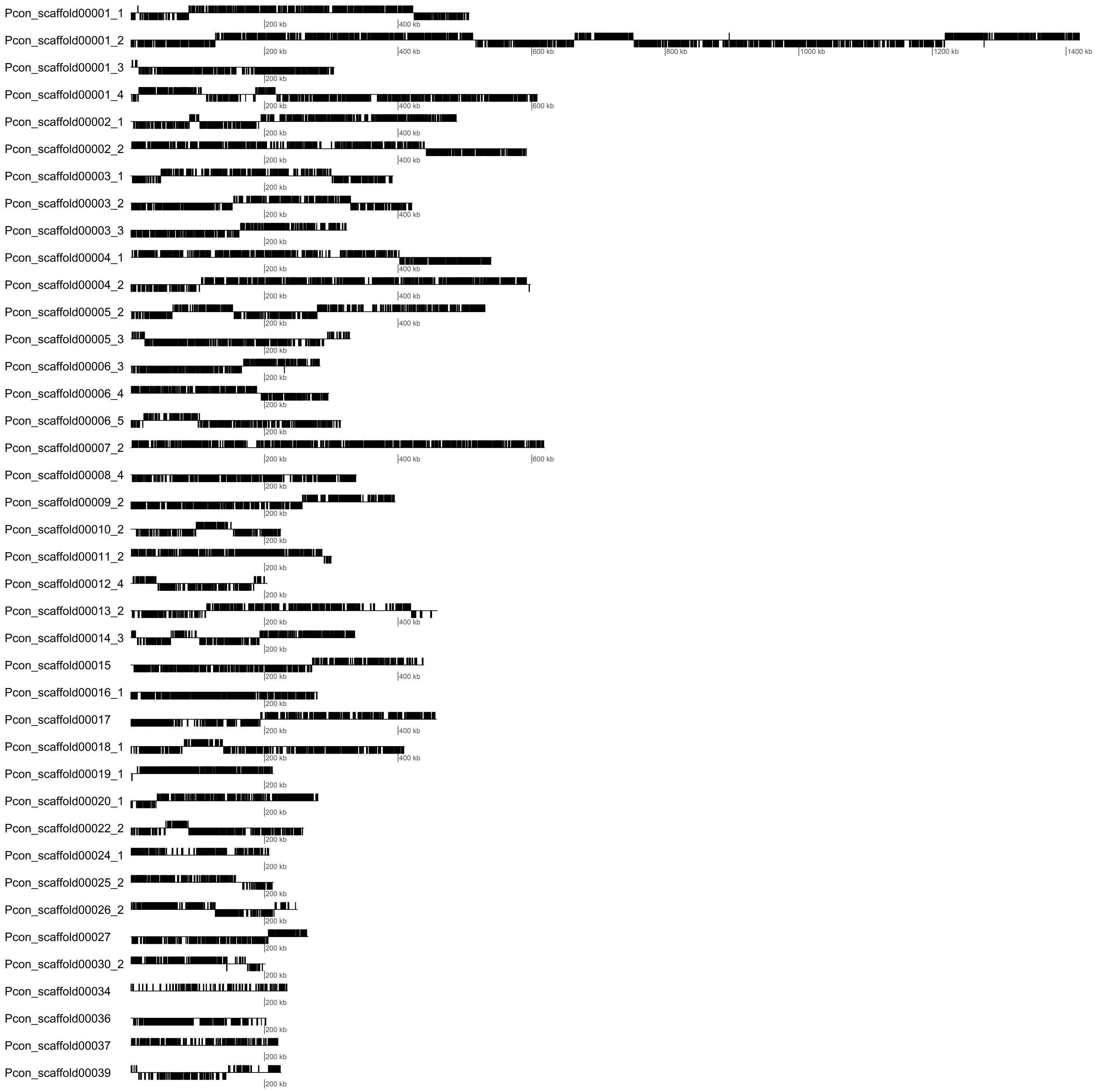
Pcon_scaffold00001_1
Pcon_scaffold00001_2
Pcon_scaffold00001_3
Pcon_scaffold00001_4
Pcon_scaffold00002_1
Pcon_scaffold00002_2
Pcon_scaffold00003_1
Pcon_scaffold00003_2
Pcon_scaffold00003_3
Pcon_scaffold00004_1
Pcon_scaffold00004_2
Pcon_scaffold00005_2
Pcon_scaffold00005_3
Pcon_scaffold00006_3
Pcon_scaffold00006_4
Pcon_scaffold00006_5
Pcon_scaffold00007_2
Pcon_scaffold00008_4
Pcon_scaffold00009_2
Pcon_scaffold00010_2
Pcon_scaffold00011_2
Pcon_scaffold00012_4
Pcon_scaffold00013_2
Pcon_scaffold00014_3
Pcon_scaffold00015
Pcon_scaffold00016_1
Pcon_scaffold00017
Pcon_scaffold00018_1
Pcon_scaffold00019_1
Pcon_scaffold00020_1
Pcon_scaffold00022_2
Pcon_scaffold00024_1
Pcon_scaffold00025_2
Pcon_scaffold00026_2
Pcon_scaffold00027
Pcon_scaffold00030_2
Pcon_scaffold00034
Pcon_scaffold00036
Pcon_scaffold00037
Pcon_scaffold00039
200 kb
400 kb
600 kb
800 kb
1000 kb
1200 kb
1400 kb

Porcisia

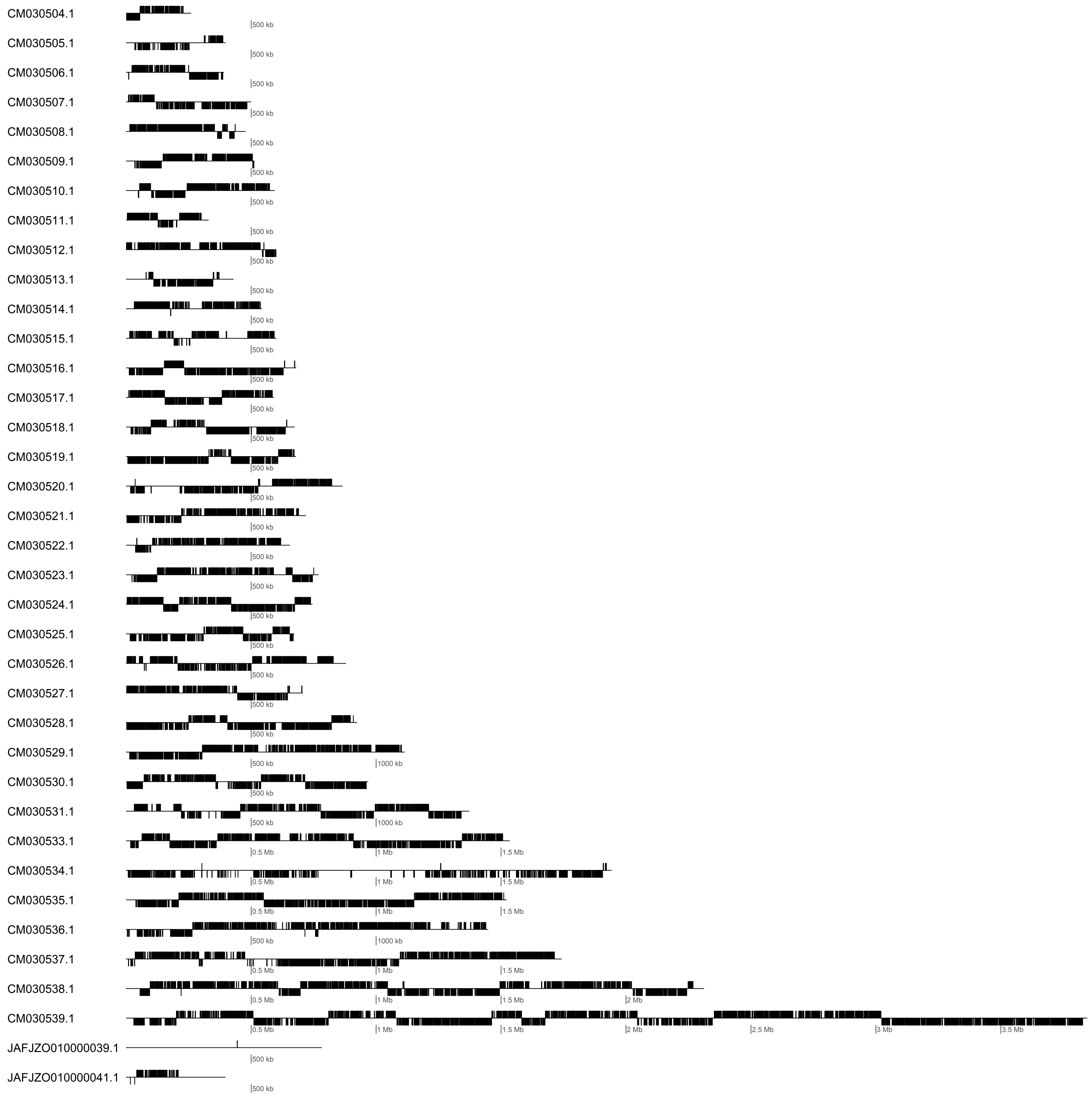
CM030504.1
CM030505.1
CM030506.1
CM030507.1
CM030508.1
CM030509.1
CM030510.1
CM030511.1
CM030512.1
CM030513.1
CM030514.1
CM030515.1
CM030516.1
CM030517.1
CM030518.1
CM030519.1
CM030520.1
CM030521.1
CM030522.1
CM030523.1
CM030524.1
CM030525.1
CM030526.1
CM030527.1
CM030528.1
CM030529.1
CM030530.1
CM030531.1
CM030533.1
CM030534.1
CM030535.1
CM030536.1
CM030537.1
CM030538.1
CM030539.1
JAFJZO010000039.1
JAFJZO010000041.1
500 kb
1000 kb
0.5 Mb
1 Mb
1.5 Mb
2 Mb
2.5 Mb
3 Mb
3.5 Mb

T. cruzi

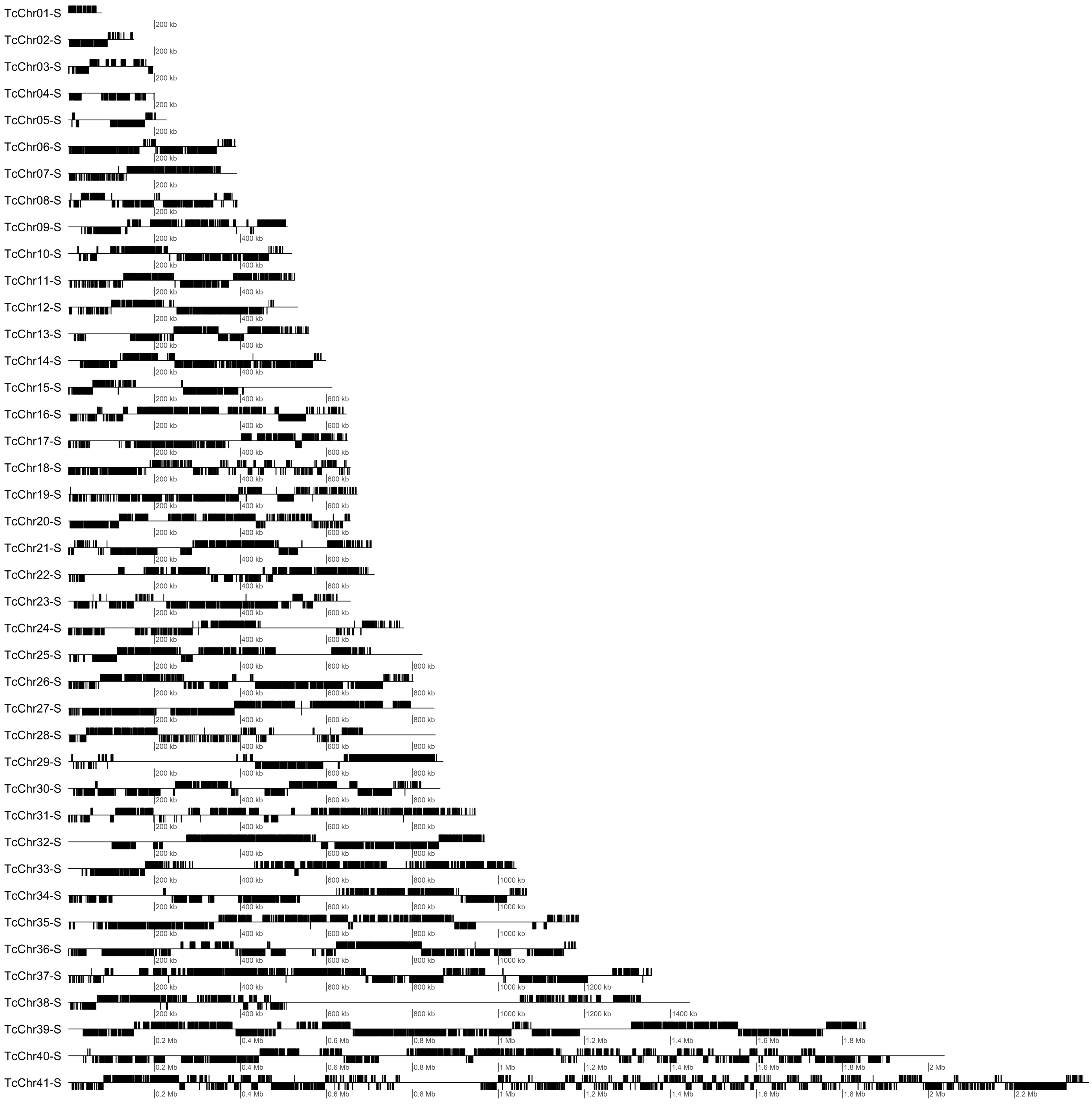
TcChr01-S
TcChr02-S
TcChr03-S
TcChr04-S
TcChr05-S
TcChr06-S
TcChr07-S
TcChr08-S
TcChr09-S
TcChr10-S
TcChr11-S
TcChr12-S
TcChr13-S
TcChr14-S
TcChr15-S
TcChr16-S
TcChr17-S
TcChr18-S
TcChr19-S
TcChr20-S
TcChr21-S
TcChr22-S
TcChr23-S
TcChr24-S
TcChr25-S
TcChr26-S
TcChr27-S
TcChr28-S
TcChr29-S
TcChr30-S
TcChr31-S
TcChr32-S
TcChr33-S
TcChr34-S
TcChr35-S
TcChr36-S
TcChr37-S
TcChr38-S
TcChr39-S
TcChr40-S
TcChr41-S
