# Supplementary material for: Ancestral aneuploidy and stable chromosomal duplication resulting in differential genome structure and gene expression control in trypanosomatid parasites
Source: Genome Res. 2024 Mar;34(3):441–53. doi: 10.1101/gr.278550.123 (PMC11067883; doi:10.1101/gr.278550.123)

Supplemental_Fig_S6.pdf: Comparison of MAF in Chr31 in *Leishmania*. A) Number of Intergenic non-synonymous and synonymous SNPs across chrs. **B)** SNP density/Kb for each chr. The red dashed line corresponds to the mean density for all chrs. Chr31 SNPs called with diploid or tetraploid SNP-calling methods are respectively in red or blue. **C)** MAF distribution in all SNP positions. The panels from the left to right correspond to: Leish Chr31 using a Tetraploid SNP caller; Leish Chr31 using a diploid SNP caller and all chrs. **D)** Evaluation of MAF in Intergenic (blue), Non-Synonym (red) or Synonym (green) positions, comparing all chrs with Leish Chr31 with both using diploid or tetraploid SNP-calling methods. **E)** MAF along Chr31 with diploid SNP-calling method, for Intergenic (blue), Non-synonymous (red) or synonymous positions (green). Each dot corresponds to a SNP position. The line corresponds to the average MAF in a sliding window of 50kb, with increments of 1kb.

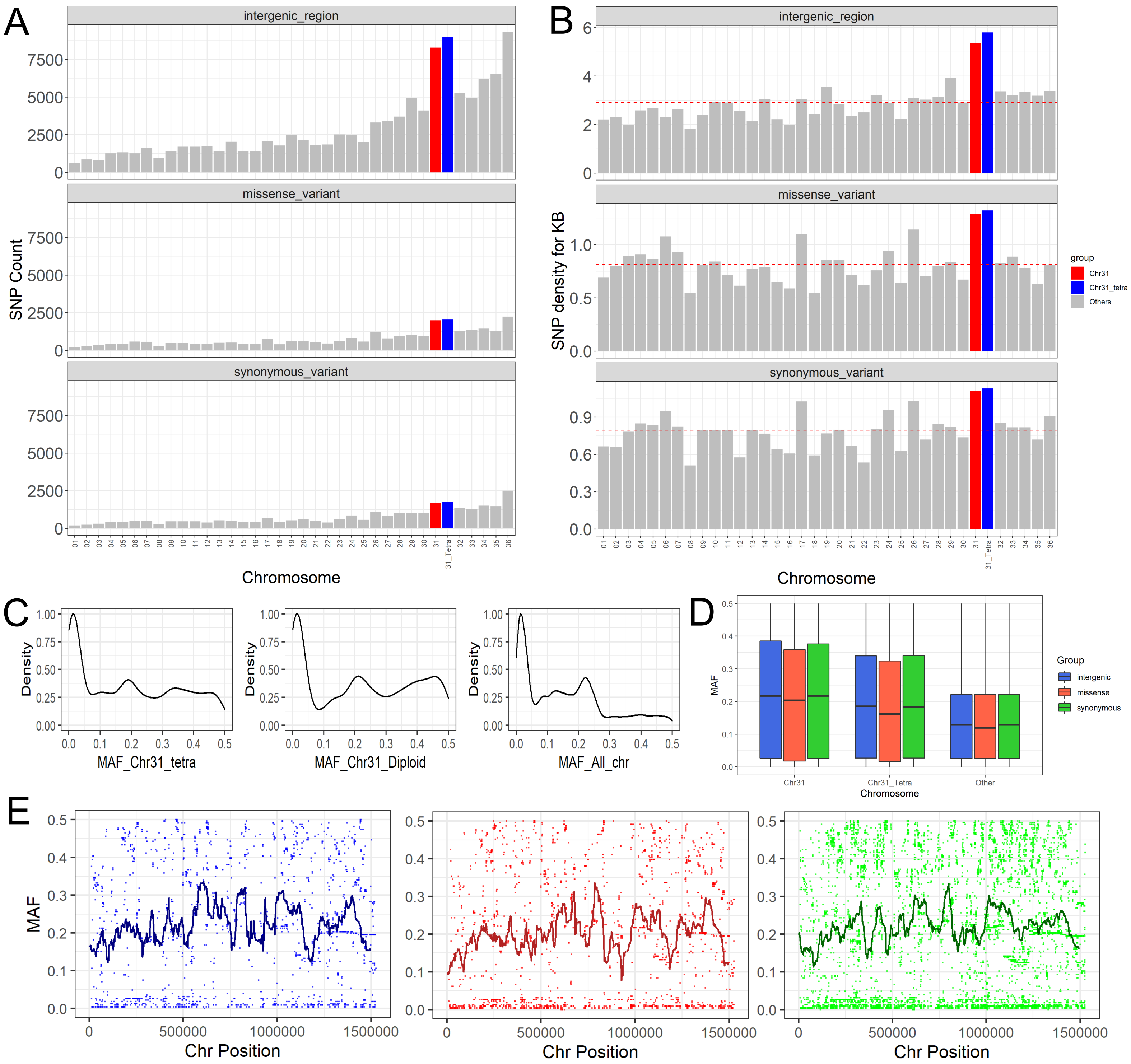

Supplement: Supplement 6 [file Supplemental_Fig_S6.pdf]
