# Supplementary material for: Ancestral aneuploidy and stable chromosomal duplication resulting in differential genome structure and gene expression control in trypanosomatid parasites
Source: Genome Res. 2024 Mar;34(3):441–53. doi: 10.1101/gr.278550.123 (PMC11067883; doi:10.1101/gr.278550.123)

Supplemental_Fig_S7.pdf: TASC have an increased nucleotide diversity that is consistent along the chromosome. Top panels correspond to box plots representing 10kb window π values comparing *TASC* with other chromosomes. Left: TASC compared to all other chromosomes. Right: π in each chromosome individually. The dashed red line corresponds to the mean π in all chromosomes. Bottom panel(s) represents the π along the chromosome/scaffolds from *TASC*, where the line represents the 10kb window π, while the dots correspond to the π value in each gene in this chromosome/scaffold. The red dashed line represents the mean π in all chromosomes. **A)** *L. donovani* EA_HIV set; **B)** *C. bombi*; **C)** *Leptomonas*.

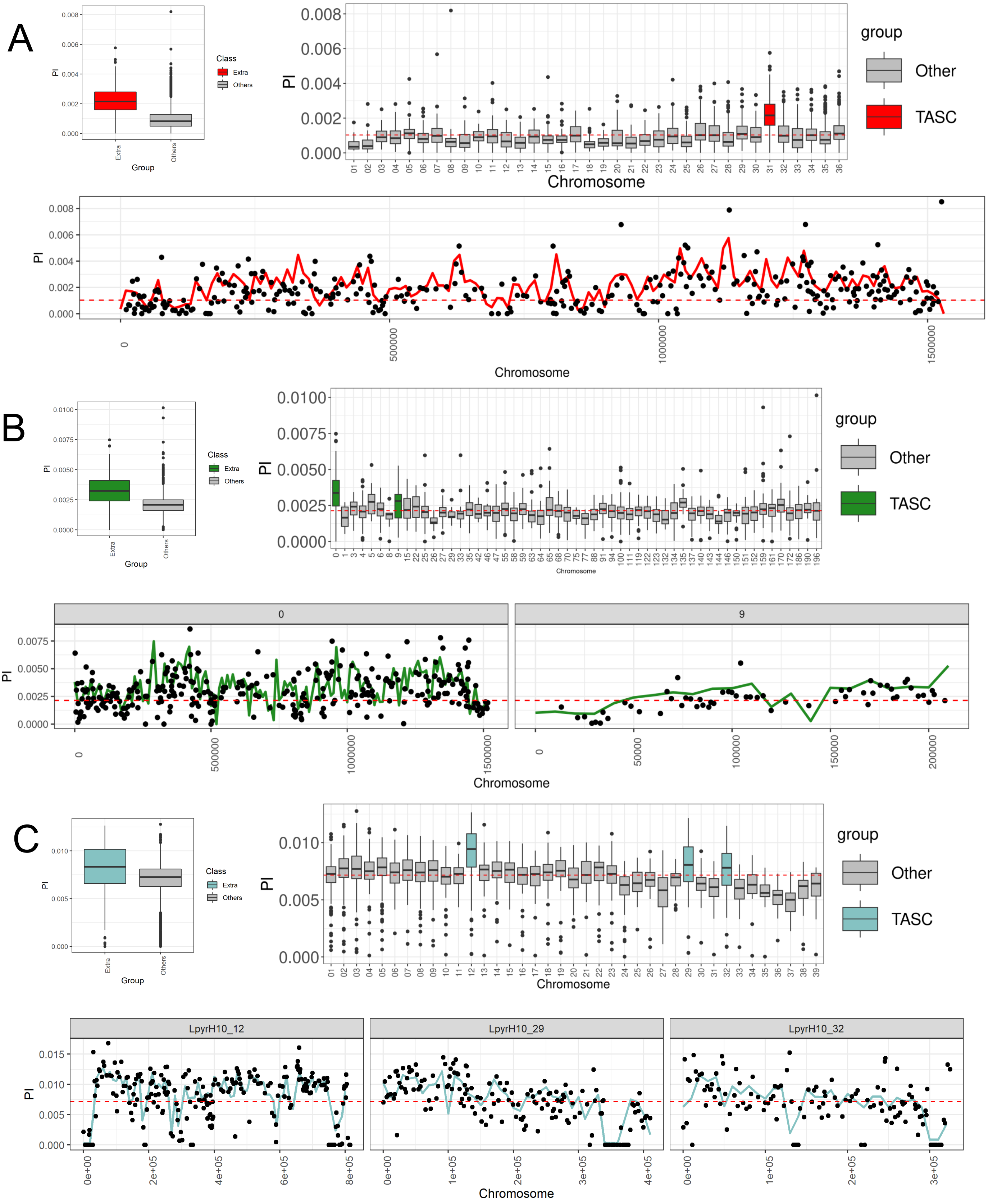

Supplement: Supplement 7 [file Supplemental_Fig_S7.pdf]
