# Supplementary material for: Ancestral aneuploidy and stable chromosomal duplication resulting in differential genome structure and gene expression control in trypanosomatid parasites
Source: Genome Res. 2024 Mar;34(3):441–53. doi: 10.1101/gr.278550.123 (PMC11067883; doi:10.1101/gr.278550.123)
Supplement: Supplement 8 [file Supplemental_Fig_S8.pdf]

Supplemental_Fig_8.pdf: High-impactful stop-codon gain in protein code genes and stop-codon loss in TASC were not informative: LdBPK.31.2.001630 pseudogene had an Alternate Allele Frequency (AF) equal to 1 in the evaluated isolates, meaning that all the isolates had the alternate allele. This suggests that this might be an error in the genome assembly or a specific alteration in the strain that was assembled, rather than a population variation. **LdLV9.31.2.207820** pseudogene has AF=0.21, however, it also has multiple internal stop codons, including stop codons upstream and downstream from the 1019617 coordinate. Hence, its biological impact was also low. **LdBPK.31.2.002230**: the mutation had a low AF (0.0132743) corresponding to only 3 heterozygous samples. It has 1257 amino acids, and the stop codon was in the position 1256, probably resulting in low biological impact. **LdBPK.31.2.002730**: the AF was even lower (AF=0.00442478) which corresponds to only one heterozygous sample. Even though this mutation has the chance of being impactful as it occurred in the aminoacid 18 in a 577 amino acids long protein, it was only observed in one sample, which hampers strong population-wise conclusions.

Chromosome	Pos	Ref	Alt	Class	Impact	Gene
**31**	362177	G	C	stop_gained	HIGH	LdBPK.31.2.000945:pseudogene
**31**	362305	C	T	stop_gained	HIGH	LdBPK.31.2.000945:pseudogene
**31**	808236	T	G	stop_lost	HIGH	LdBPK.31.2.001630:pseudogene
**31**	1019617	A	G	stop_lost	HIGH	LdLV9.31.2.207820:pseudogene
**31**	1105177	G	A	stop_gained	HIGH	LdBPK.31.2.002230
**31**	1183819	C	T	stop_gained	HIGH	LdLV9.31.2.207890:pseudogene
**31**	1336350	G	A	stop_gained	HIGH	LdBPK.31.2.002730
