# Supplementary material for: Ancestral aneuploidy and stable chromosomal duplication resulting in differential genome structure and gene expression control in trypanosomatid parasites
Source: Genome Res. 2024 Mar;34(3):441–53. doi: 10.1101/gr.278550.123 (PMC11067883; doi:10.1101/gr.278550.123)

Supplemental_Fig_S9.pdf: Correlation between CCNV and gene expression. A) Correlation between the DNA copies (X axis) and RNA expression (Y axis) of all 36 Leishmania chrs in 7 clones (r=0.647994, p-value 4.14e-27). Leish Chr31 is highlighted in red. This image is a confirmation of the results described in Barja 2017 (Prieto Barja et al. 2017). DNA (red line) and RNA (blue line) AARD along Leish Chr31 in C3 **(B)**; C4 **(C)**; C6 **(D)**; C7**(E)**; C8 **(F)**; C9 **(G)** and C10 **(H)**.

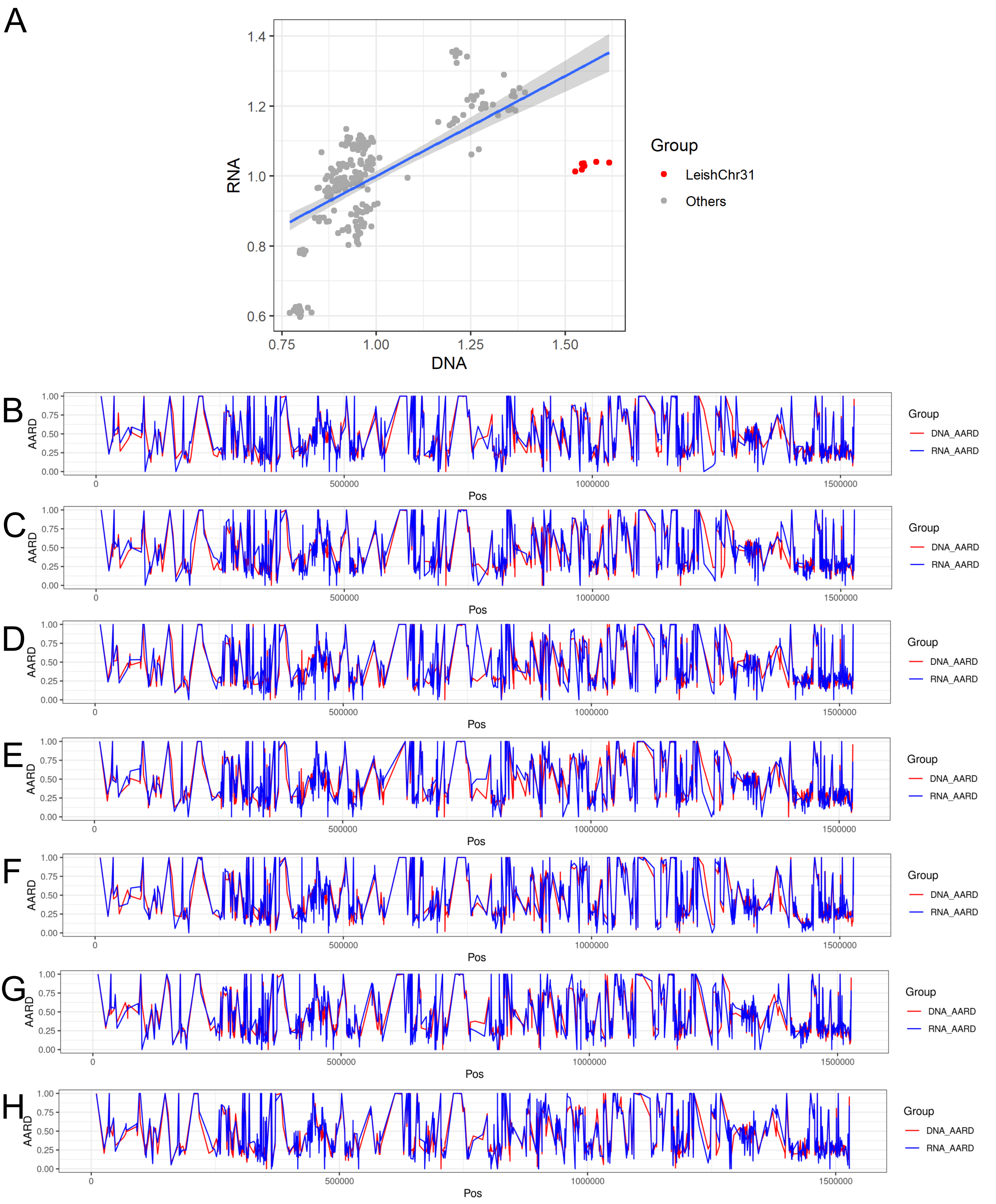

Supplement: Supplement 9 [file Supplemental_Fig_S9.pdf]
