# Supplementary material for: Ancestral aneuploidy and stable chromosomal duplication resulting in differential genome structure and gene expression control in trypanosomatid parasites
Source: Genome Res. 2024 Mar;34(3):441–53. doi: 10.1101/gr.278550.123 (PMC11067883; doi:10.1101/gr.278550.123)

Supplemental_Fig_S10.pdf: Codon usage in *L. donovani* genes in each chromosome. Comparison of Measure Independent of Length and Composition (MILC) in Chromosome 31 (red) and all other chromosomes combined (black), represented as **A)** density plots, **B)** boxplot and violin plots. **C)** Comparison of MILC (Y axis) and gene length (X axis), D) Log10 MILC in each chromosome. E) Heatmap of the codon relative presence in genes from each chromosome.

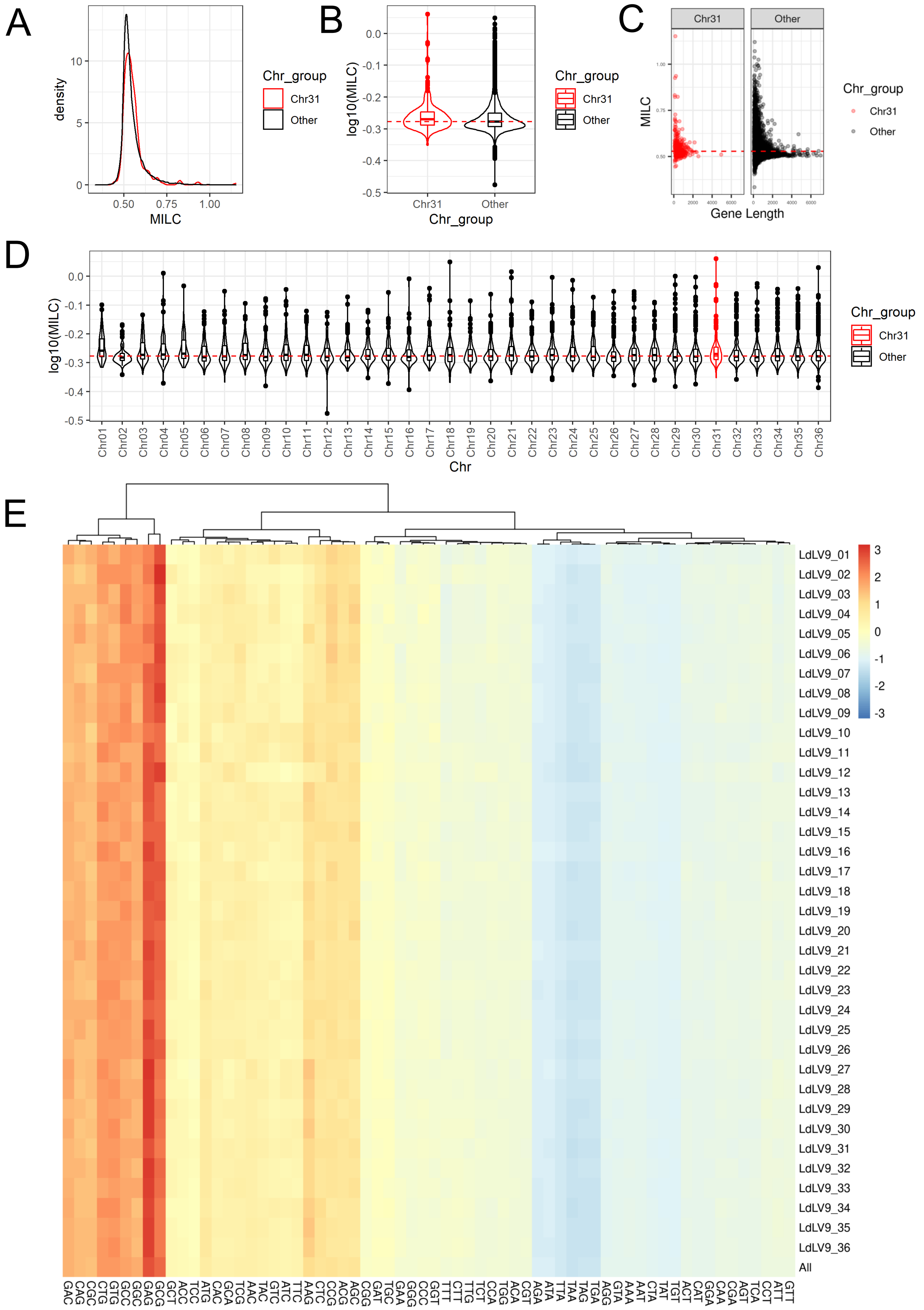

Supplement: Supplement 10 [file Supplemental_Fig_S10.pdf]
